# Cardiac progenitor cell-derived extracellular vesicles promote angiogenesis through both associated- and co-isolated proteins

**DOI:** 10.1038/s42003-023-05165-7

**Published:** 2023-08-01

**Authors:** Marieke Theodora Roefs, Julia Bauzá-Martinez, Simonides Immanuel van de Wakker, Jiabin Qin, Willem Theodoor Olijve, Robin Tuinte, Marjolein Rozeboom, Christian Snijders Blok, Emma Alise Mol, Wei Wu, Pieter Vader, Joost Petrus Gerardus Sluijter

**Affiliations:** 1 Department of Experimental Cardiology, University Medical Center Utrecht, Utrecht University, Utrecht, The Netherlands; 2 Biomolecular Mass Spectrometry and Proteomics, Bijvoet Center for Biomolecular Research and Utrecht Institute for Pharmaceutical Sciences, Utrecht University, Utrecht, The Netherlands; 3 Singapore Immunology Network (SIgN), ASTAR (Agency for Science, Technology and Research), Singapore, Singapore; 4 CDL Research, University Medical Center Utrecht, Utrecht, The Netherlands

**Keywords:** Extracellular signalling molecules, Heart stem cells, Stem-cell therapies

## Abstract

Extracellular vesicles (EVs) are cell-derived lipid bilayer-enclosed particles that play a role in intercellular communication. Cardiac progenitor cell (CPC)-derived EVs have been shown to protect the myocardium against ischemia-reperfusion injury via pro-angiogenic effects. However, the mechanisms underlying CPC-EV-induced angiogenesis remain elusive. Here, we discovered that the ability of CPC-EVs to induce in vitro angiogenesis and to stimulate pro-survival pathways was lost upon EV donor cell exposure to calcium ionophore. Proteomic comparison of active and non-active EV preparations together with phosphoproteomic analysis of activated endothelial cells identified the contribution of candidate protein PAPP-A and the IGF-R signaling pathway in EV-mediated cell activation, which was further validated using in vitro angiogenesis assays. Upon further purification using iodixanol gradient ultracentrifugation, EVs partly lost their activity, suggesting a co-stimulatory role of co-isolated proteins in recipient cell activation. Our increased understanding of the mechanisms of CPC-EV-mediated cell activation will pave the way to more efficient EV-based therapeutics.

## Abstract (web summary)

Cardiac progenitor cell-derived extracellular vesicles (EVs) are characterized by proteomics and found to induce angiogenesis in a manner dependent on both EV-associated and co-isolated proteins, including Pappalysin-1.

## Introduction

Myocardial infarction causes massive loss of cardiomyocytes, resulting in scar formation and cardiac remodelling which lead to impaired cardiac function and progressively into development of heart failure. Although heart failure cannot be prevented by currently available therapies, recent animal studies have demonstrated that cardiac function post-myocardial infarction may be improved by the therapeutic application of stem- and cardiac progenitor cell (CPC)-derived extracellular vesicles (EVs)^1,2^.

EVs are cell-derived nanoparticles enclosed by a lipid bilayer that contain biological cargo including RNA, proteins and lipids and play a role in normal cellular homoeostasis and intercellular communication^3^. EVs have the ability to activate target cells through the presence of adhesion molecules and receptors and via the delivery of bioactive molecules derived from the parent cell^2,4^. After in vivo administration, EVs released by Sca+ CPCs modulate regenerative processes in the heart by promoting angiogenesis, decreasing fibrosis and inhibiting cardiomyocyte apoptosis and thereby contributing to cardiac repair^5,6^. EVs derived from other stem cell sources have been shown to deliver distinct miRNAs and proteins to different cells in the heart to promote cardiac recovery^7–9^. Despite attempts to document CPC-EV composition, there remains a lack of functional and mechanistic studies elucidating exactly which components in the complex protein repertoire are responsible for reparative function. Moreover, clinical application of stem cell-derived EVs is hampered by reproducibility issues related to differences in therapeutic activity between different EV isolations, among others^10,11^. Size-exclusion chromatography (SEC) has been widely adopted as a preferable method for EV isolation^12,13^. However, as for most methods to date, it does not yield a completely pure EV population. It has been speculated that co-isolated proteins present in EV preparations may contribute to their therapeutic function^14–18^. Therefore, deeper functional characterization of the CPC-EV content, and localization of this content in CPC-EV preparations is needed to get better insights into the mechanisms of action leading to a more reproducible therapeutic application of CPC-EVs. In this study, we set out to unravel the protein-mediated effects of CPC-EVs on endothelial cells. First, we identified the functional protein components of CPC-EVs involved in human microvascular endothelial cell (HMEC-1) activation by comparing the content of functional and non-functional (CPC-) EV preparations. Next, we studied the contribution of individual EV-associated proteins Pregnancy-associated plasma protein A (PAPP-A) and Nidogen-1 (NID1) to CPC-EV-mediated angiogenesis by the generation of knock-out (KO) EVs employing CRISPR/Cas9 technology. Lastly, we investigated the contribution of EV-associated versus co-isolated proteins to CPC-EV function by employing iodixanol gradient density-based purification.

## Results

### CPC-EVs activate HMEC-1 and induce HMEC-1 migration

**Fig. 1:**
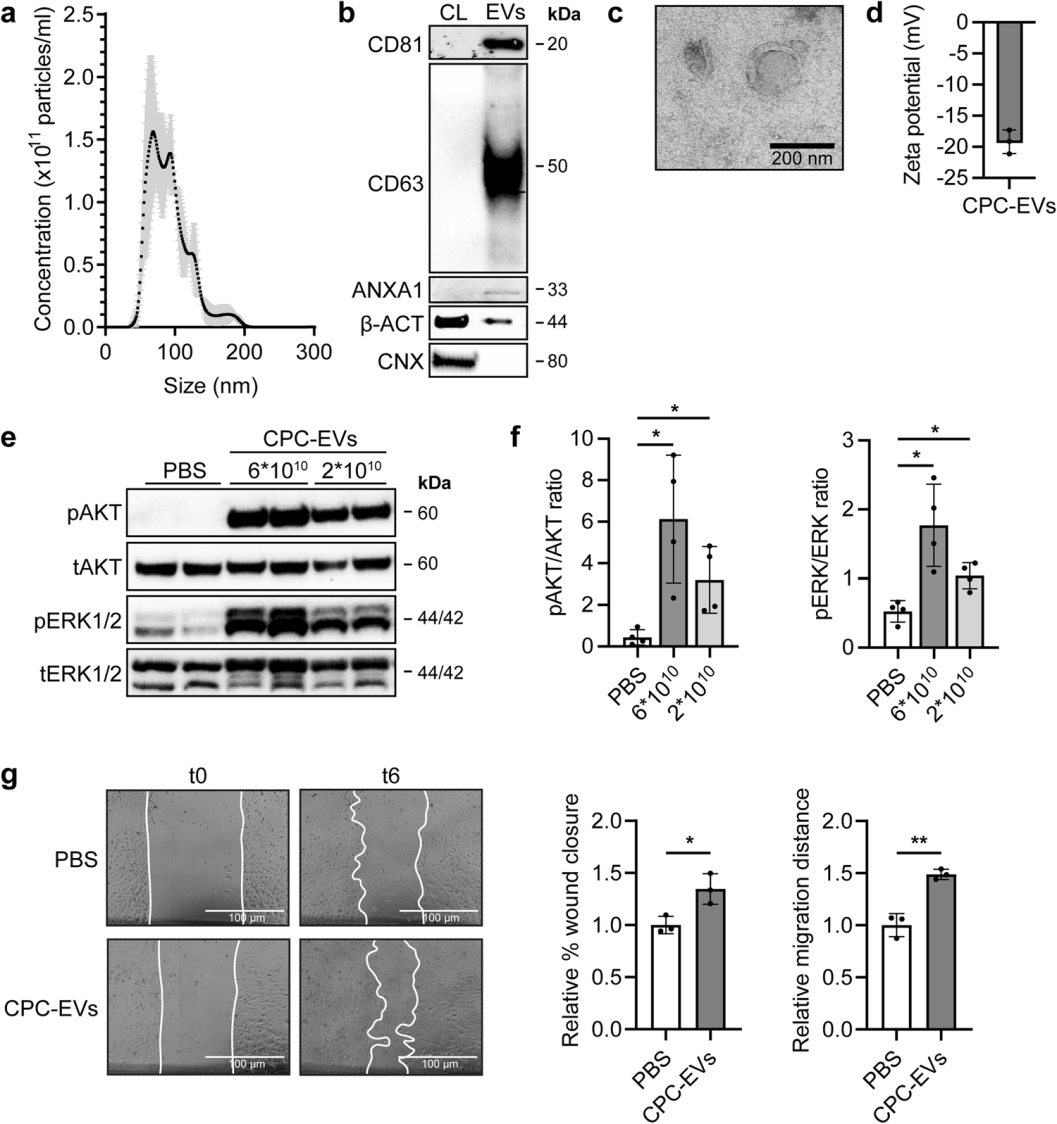
CPC-EVs activate intracellular signalling in HMEC-1 and induce HMEC-1 migration. **a** Representative NTA plot showing the size distribution and particle concentration of EVs isolated from the conditioned medium of CPCs (CPC-EVs) using size-exclusion chromatography. **b** Western blot analysis showing the presence of CD81, CD63, Annexin A1 (ANXA1), β-actin (β-ACT), and absence of Calnexin (CNX) in CPC-EVs. β-ACT and CNX were present in CPC lysate (CL). **c** Representative TEM image of CPC-EVs. **d** Surface charge (zeta potential) of CPC-EVs as measured by laser Doppler electrophoresis. **e**, **f** Representative western blot analysis of phosphorylated AKT (pAKT), total AKT (tAKT), phosphorylated ERK1/2 (pERK1/2) and total ERK1/2 (tERK1/2) in HMEC-1 treated with two different CPC-EV particle doses (dosing based on ref. ^12^). **f** Quantification of pAKT, tAKT, pERK1/2 and tERK1/2 expression levels using densitometry expressed as pAKT/AKT and pERK/ERK ratios (*n* = 4). **g** Wound healing assay showing effects of CPC-EVs on HMEC-1 migration 6 h (t6) after EV addition (t0), analysed both as relative % wound closure and relative absolute migration distance compared to PBS (*n* = 3). Data are presented as mean ± SD. *p < 0.033; **p < 0.0021.

In the heart, pro-reparative effects evoked by the administration of CPCs and CPC-derived EVs were shown to be mediated by promoting angiogenesis^19–21^. To confirm the pro-angiogenic potential of our CPC-EVs in vitro, EVs were isolated from serum-free conditioned medium using SEC as described previously^12^. Successful isolation of CPC-EVs was confirmed by NTA which demonstrated a size distribution with a peak at ~90 nm (Fig. 1a). Western blot, according to MISEV2018 guidelines^22^, confirmed the relative enrichment of EV marker proteins CD81, CD63, and Annexin A1 in EVs as compared to cell lysate and absence of endoplasmatic reticulum protein Calnexin (Fig. 1b). Transmission electron microscopy (TEM) of the CPC-EV preparation demonstrated the presence of vesicles containing a double leaflet membrane (Fig. 1c). The surface charge (zeta potential) of EVs was negative, −19.3 mV, as measured by laser Doppler electrophoresis (Fig. 1d).

Previous studies have demonstrated activation of the mitogen-activated protein kinase1/2 (MAPK1/2)-extracellular signal-regulated kinase1/2 (ERK1/2) and PI3 Kinase/AKT signalling pathways in HMEC-1 upon CPC-EV administration^12^. Indeed, levels of AKT and ERK1/2 phosphorylation increased within 30 min after addition of CPC-EVs in a dose-dependent manner (Fig. 1e, f). The capacity of CPC-EVs to functionally activate endothelial cells was determined in a HMEC-1 scratch wound assay. Addition of 2 × 10^10^ CPC-EVs to scratched HMEC-1 induced cell migration as determined by both percentage of closure and absolute migration distance compared to PBS (Fig. 1g), while not affecting total cell number as determined by total protein content in HMEC-1 lysates (Supplementary Fig. 1a). This validated the pro-angiogenic activity of CPC-EVs in vitro.

### Endothelial cell-activating capacity of CPC-EVs is lost after CPC exposure to calcium ionophore

**Fig. 2:**
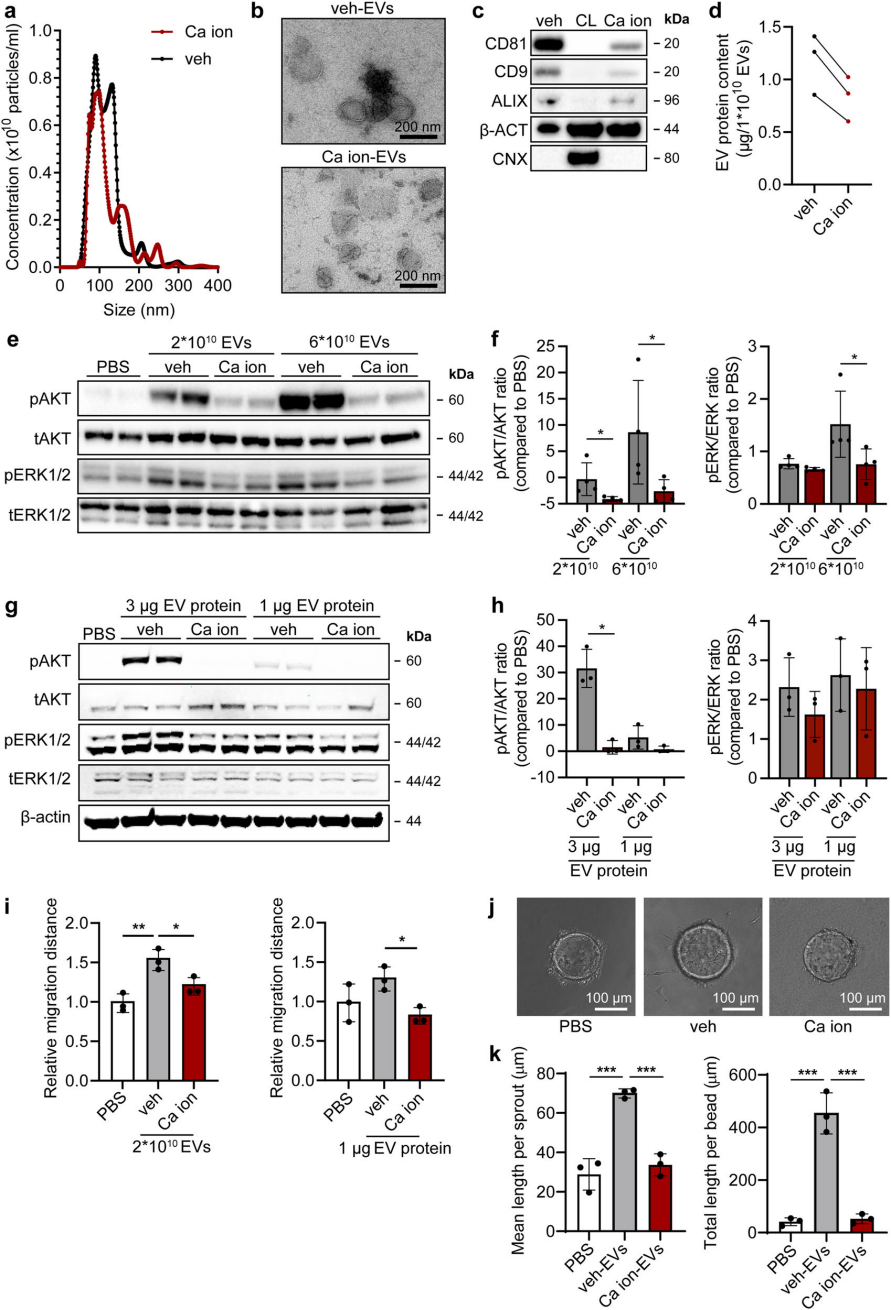
EVs isolated from CPCs activate intracellular signalling in HMEC-1 and induce HMEC-1 migration, which is lost after CPC exposure to calcium ionophore. **a** Representative NTA plot showing the size distribution and particle concentration of EVs isolated from the same volume of vehicle- (0.0125% DMSO; veh-EVs) or calcium ionophore- (Ca ion-EVs) stimulated CPCs. **b** Representative TEM images of veh- and Ca ion-EVs. (**c**) Western blot analysis showing the presence of CD81, CD9, ALIX, β-actin (β-ACT), and absence of Calnexin (CNX) in veh- and Ca ion-EVs. β-ACT and CNX were present in CPC lysate (CL). **d** Protein content per 1 × 10^10^ veh- and Ca ion-EVs of three biological triplicates. **e**–**h** Representative western blot analysis of phosphorylated AKT (pAKT), total AKT (tAKT), phosphorylated ERK1/2 (pERK1/2) and total ERK1/2 (tERK1/2) in HMEC-1 treated with veh- and Ca ion-EVs normalized on two doses of (**e**, **f**) EV particle numbers or (**g**, **h**) EV total protein content. β-ACT was included as housekeeping protein. **f**, **h** Quantification of pAKT, tAKT, pERK1/2 and tERK1/2 expression levels using densitometry expressed as pAKT/AKT and pERK/ERK ratios (*n* = 4 and *n* = 3). Biological replicates of (**e**, **g**) are also displayed in Supplementary Fig. 9a–c. **i** Wound healing assay showing effects of 2 × 10^10^ or 1 µg veh- and Ca ion-EVs on HMEC-1 migration (*n* = 3). **j** Sprouting assay showing veh- and Ca ion-EV-induced HMEC-1 sprout formation on beads, analysed both as (**k**) mean length per sprout and total sprout length per bead (*n* = 3, technical replicates. Data are representative of two biologically independent experiments). Data are presented as mean ± SD. *p < 0.033; **p < 0.0021, ***p < 0.0002.

In order to better investigate the pro-migratory CPC-EV content, we first set out to induce CPC-EV release. Calcium ionophore A23187 has been described to stimulate EV release through increasing intracellular calcium levels^23–26^. To investigate calcium ionophore-induced EV release by CPCs, CPCs were either left untreated (0.0125% DMSO, veh-EVs) or exposed to 1 µM calcium ionophore (Ca ion-EVs) for 24 h. EVs were then isolated from serum-free conditioned medium using SEC. Size and total number of released EVs were not affected upon CPC exposure to calcium ionophore, as determined by NTA (Fig. 2a). TEM confirmed the presence of round, membrane enclosed particles in both EV preparations (Fig. 2b). Both EV isolates displayed EV marker proteins CD81, CD9 and ALIX, although their presence was reduced in Ca ion-EVs (Fig. 2c). Ca ion-EVs had a slightly lower total protein content per 1 × 10^10^ particles compared to veh-EVs (Fig. 2d). Therefore, in subsequent experiments, to prevent bias in interpreting EV functionality caused by differences in EV purity, EV supplementation was normalized between conditions both on total particle number and on total protein amount. Notably, veh-EV stimulation induced AKT and ERK1/2 phosphorylation in HMEC-1, while Ca ion-EV stimulation did not, both when EV addition was normalized based on particle number (Fig. 2e, f, Supplementary Fig. 9a, b) and on total protein levels (Fig. 2g, h, Supplementary Fig. 9c). EVs released from SKOV-3 cells were simultaneously isolated and characterized as they were previously demonstrated to be unable to induce AKT and ERK1/2 signalling (Supplementary Fig. 1b, c). Indeed, SKOV-3-EVs were ineffective in inducing AKT and ERK1/2 phosphorylation as compared to veh-EVs (Supplementary Fig. 1d–g) and were therefore included as non-functional non-stem cell control. Furthermore, veh-EV stimulation, but not Ca ion-EV stimulation, induced HMEC-1 migration in a wound closure scratch assay (Fig. 2i) and stimulated sprout formation in an HMEC-1 sprouting assay (Fig. 2j, k). These differences in EV functionality suggest that calcium ionophore treatment, although not increasing total EV release, affects EV content. This finding to isolate both functional and non-functional EV populations from CPCs was used to further explore the contributing signalling pathways involved in CPC-EV-mediated cell activation.

### Phosphoproteome analysis identifies specific signalling pathways involved in CPC-EV-mediated HMEC-1 activation

**Fig. 3:**
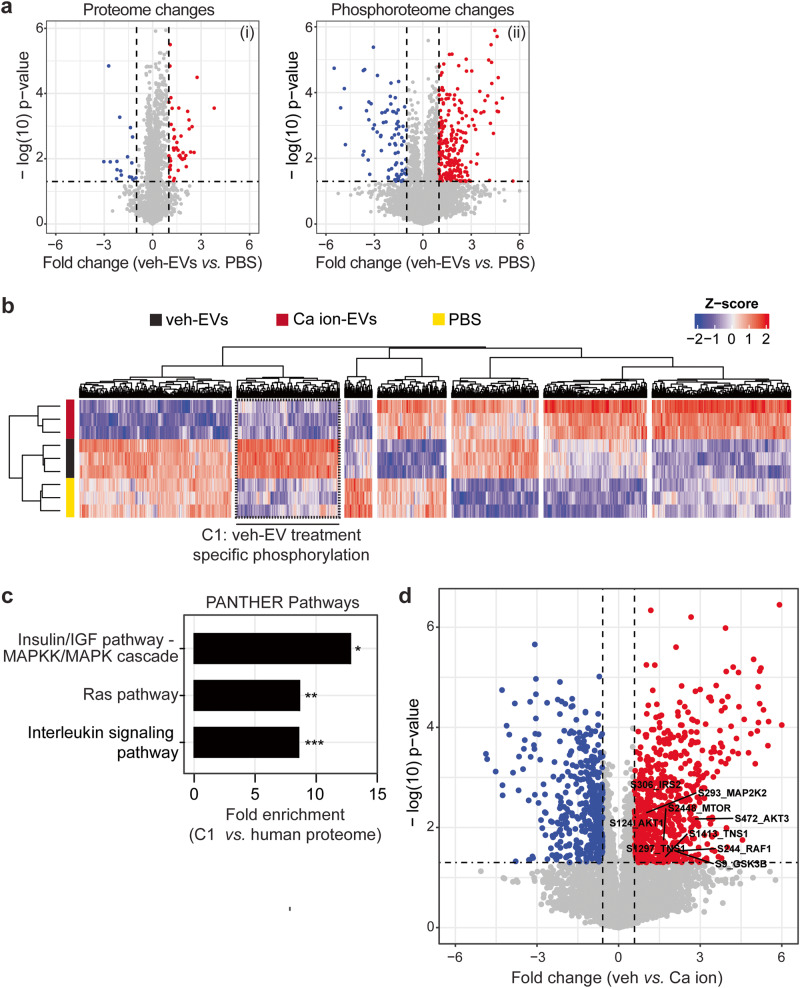
Phosphoproteomic analysis of HMEC-1 upon veh-EV- and Ca ion-EV stimulation. **a** Volcano plots showing changes in the (i) proteome and (ii) phosphoproteome of HMEC-1 after veh-EV stimulation compared to negative control (PBS). *P*-values were calculated using student’s T-test, and significantly changing proteins (*p*-value ≤ 0.05 and fold change >2) in veh-EV-treated HMEC-1 are highlighted in red, while significantly changing proteins in PBS are highlighted in blue. **b** Hierarchical clustering of 1549 significantly changing phosphosites (ANOVA, *q*-value ≤ 0.05) found in HMEC-1 upon stimulation with veh-EVs, Ca ion-EVs and PBS. Cluster C1, including veh-EV-induced specific phosphorylation, is highlighted with dashed lines. **c** PANTHER Pathway enrichment analysis of phosphoproteins found in clusters C1, ranked on fold enrichment. *= FDR < 0.05, **= FDR < 0.01, ***= FDR < 0.005. **d** Volcano plot showing fold changes in the phosphoproteome of HMEC-1 upon veh-EV compared with Ca ion-EV stimulation, also represented in Fig. 6a. *P*-values were calculated using student’s T-test, and significantly changing phosphosites (*p*-value < 0.05 and fold change >2) after veh-EV treatment are highlighted in red, while significantly changing phosphosites after Ca ion-EV treatment are highlighted in blue.

To get insights into the mechanism of CPC-EV-mediated cell activation, the signal transduction pathways triggered upon HMEC-1 stimulation with functional veh-, non-functional Ca ion-EVs and negative control (PBS) were characterized by phosphoproteomics (Supplementary Fig. 2). When comparing veh-EV- to PBS-treated HMEC-1 cells after 30 min of stimulation, significant changes were observed specifically at the phosphoproteome level, independent of proteome regulation (Fig. 3a). The phosphoproteome analyses were highly sensitive, identifying ~6900 class-I phosphosites (with phospho-modification localisation probability >0.75) from only 50 µg of HMEC-1 lysates. Hierarchical clustering of the significantly changing phosphosites revealed one cluster with increased phosphorylation in veh-EV-treated HMEC-1 compared to PBS- and Ca ion-EV-treated HMEC-1 (Fig. 3b, dashed cluster C1). To better understand which intracellular signalling pathways were activated by veh-EVs, the phosphosites present in cluster C1 (*n* = 195) were further annotated against PANTHER pathways. The Insulin/IGF pathway-MAPKK/MAPK cascade, Ras- and Interleukin signalling pathways were identified as the most enriched pathways in HMEC-1 (Fig. 3c). Furthermore, significantly altered phosphosites between veh- and Ca ion-EV-stimulated HMEC-1 included members of the PI3K-AKT and MAPK signalling pathways (highlighted in Fig. 3d). This demonstrates the specific intracellular pathways implicated in CPC-EV-mediated HMEC-1 activation.

### Removal of surface proteins from EVs by proteinase K treatment decreases EV functionality on endothelial cells

**Fig. 4:**
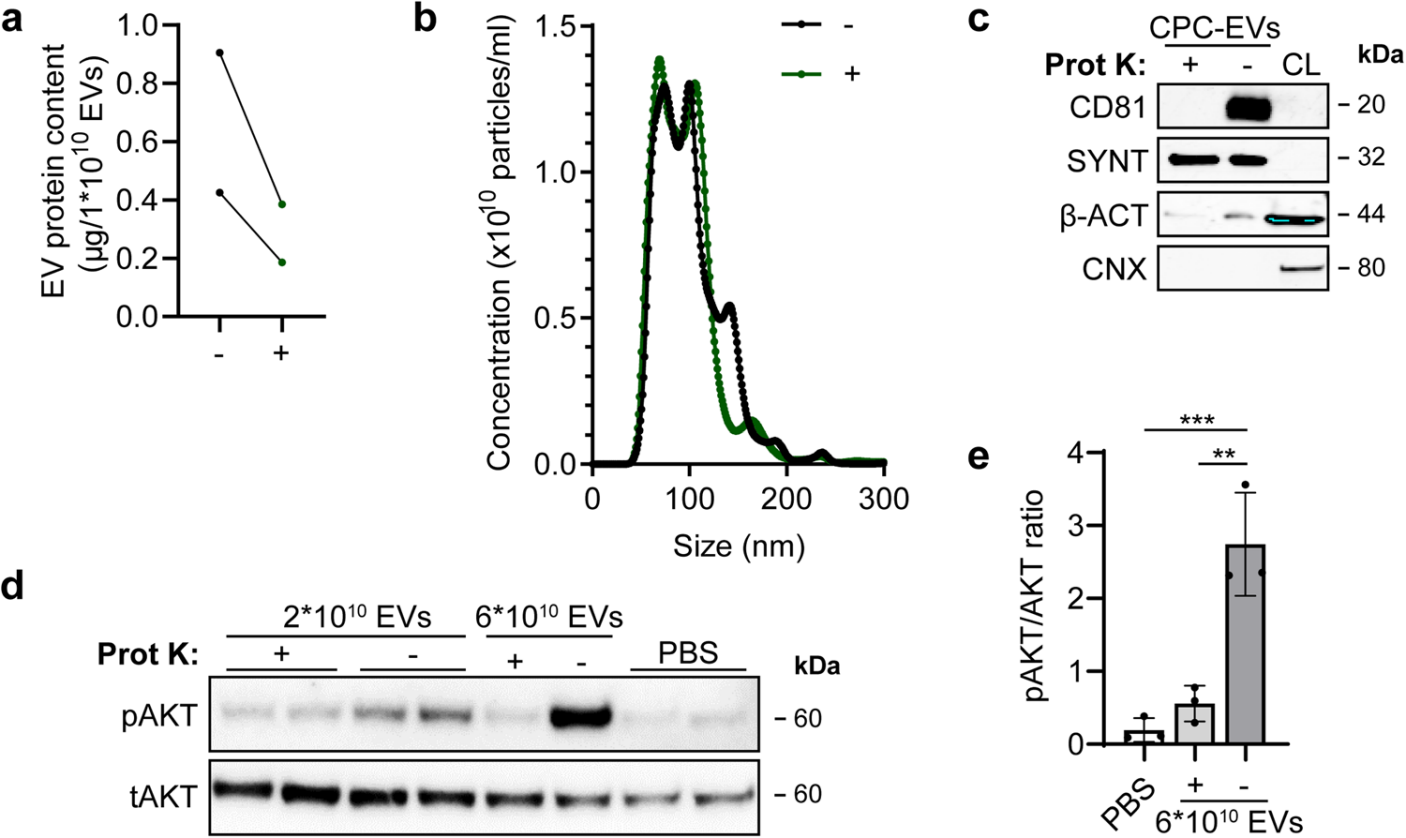
CPC-EVs lose HMEC-1 activating capacity after Proteinase K treatment. **a** Protein content per 1 × 10^10^ EVs treated with (+) and without (-) Proteinase K (Prot K) of two representative experiments. **b** Representative NTA plot showing the size distribution and particle concentration of both EV populations after a second SEC isolation. **c** Western blot analysis showing the absence and presence of CD81 in Prot K-treated (+) and untreated (-) EVs, respectively. Syntenin-1 (SYNT) and β-actin (β-ACT) were present in both EV populations, while absent for Calnexin (CNX). β-ACT and CNX were present in CPC lysate (CL). **d** Representative western blot analysis of phosphorylated AKT (pAKT) and total AKT (tAKT) in HMEC-1 stimulated with Prot K- (+) and untreated (-) CPC-EVs normalized on two doses of EV particle numbers. **e** Quantification of pAKT and tAKT, expression levels using densitometry expressed as pAKT/AKT ratio (*n* = 3). Biological replicate of (**d**) is displayed in Supplementary Fig. 9d. Data are presented as mean ± SD. **p < 0.0021, ***p < 0.0002.

CPC-EVs induce activation of intracellular signalling within 30 min after administration to HMEC-1 (Figs. 1e, 3a), which suggests a role for direct receptor-ligand interactions of EVs with the target cell membrane. To determine the contribution of EV-associated proteins to CPC-EV functionality, CPC-EV surface proteins and the extracellular receptor domains of transmembrane proteins were removed via treatment with 100 µg/mL proteinase K. Proteinase K-treated EVs and untreated EVs subsequently underwent a second SEC separation to remove free protein fragments from EVs. A reduction in protein levels in proteinase K-treated EVs was verified with microBCA analysis (Fig. 4a), and NTA demonstrated that EV size was not affected by proteinase K treatment (Fig. 4b). Western blot analyses confirmed the removal of EV-surface protein CD81 in proteinase K-treated EVs, while intravesicular protein Syntenin-1 was unaffected (Fig. 4c). Proteinase K-treated EVs were less potent to induce phosphorylation of AKT in HMEC-1 as compared to untreated EVs (Fig. 4d, e, Supplementary Fig. 9d), which confirmed a major contribution of CPC-EV-associated proteins to HMEC-1 activation.

### Proteomic analysis of CPC-EVs identifies proteins involved in stimulation of endothelial cell migration

**Fig. 5:**
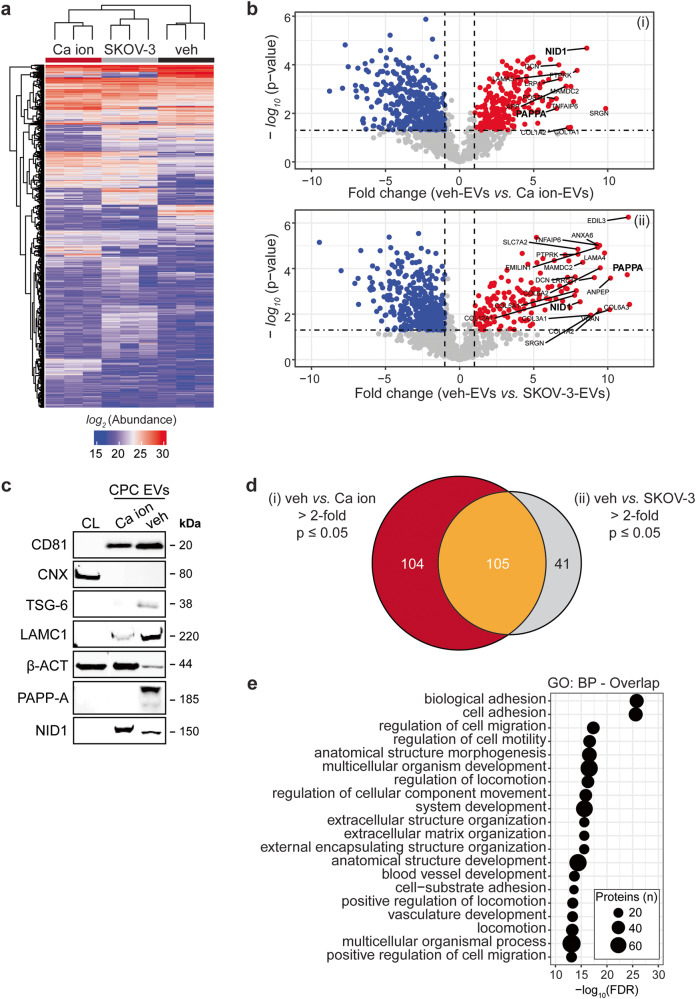
LC–MS/MS identified enriched proteins in veh-EVs compared with Ca ion- and SKOV-3-EVs. **a** Heat map of protein abundance (log_2_) of proteins identified in each biological replicate (veh-, Ca ion- and SKOV-3-EVs), as identified by LC–MS/MS. **b** Volcano plots showing average fold changes for protein abundance (log_2_) of proteins identified in veh-EVs compared to (i) Ca ion-EVs and (ii) SKOV-3-EVs. *P*-values were calculated using student’s T-test, and significantly changing proteins (*p*-value ≤ 0.05 and fold change >2) in veh-EVs are highlighted in red, while significantly changing proteins in Ca ion- and SKOV-3-EVs are highlighted in blue. **c** Western blot analysis confirming the enrichment of MS-identified proteins NID1, TSG-6, LAMC1, PAPP-A, CD81 and β-actin (β-ACT) in veh-EVs compared with Ca ion-EVs. CNX was solely present in CPC lysate (CL). Complete blots of β-ACT, PAPP-A and NID1 are displayed in Supplementary Fig. 10. **d** Venn diagram showing number of proteins with >2-fold significant enrichment (*p* ≤ 0.05) in veh-EVs compared to Ca ion- and SKOV-3-EVs, and overlap between those two populations. **e** Gene ontology analysis using PANTHER of enriched biological processes for the 105 overlapping proteins, depicting number of identified proteins in each group, ranked on smallest corrected *p*-value (−log_10_(FDR)).

We next sought to identify the CPC-EV proteins involved in the activation of intracellular signalling and HMEC-1 migration, by employing the differences in functionality between veh-, Ca ion- and SKOV-3-EVs. Identification of differentially expressed proteins between functional veh-EVs and non-functional Ca ion-EVs released from the same CPC donor eliminates differentially expressed proteins due to differences in cell donor, while SKOV-3 EVs were included as additional non-functional non-stem cell control. Using an unbiased label-free proteomics strategy, we compared quantitatively the total EV proteome between the three sources. From this 2077 proteins were confidently identified (1% FDR) amongst all EV populations. From these, 1293 proteins were detected in at least 2 out of the 3 biological replicates for each EV population (Fig. 5a), of which we further explored the 209 proteins that were significantly altered between veh-EVs and Ca ion-EVs (Fig. 5b, i), and 146 proteins between veh-EVs and SKOV-3-EVs (Fig. 5b, ii). The enrichment of PAPP-A, tumour necrosis factor-stimulated gene-6 (TSG-6) and Laminin subunit gamma 1 (LAMC1) in veh-EVs as compared to Ca ion-EVs was validated by western blotting (Fig. 5c).

Further analysis of the significantly enriched proteins in veh-EVs revealed 105 proteins consistently enriched in veh-EVs compared to both the Ca ion- and SKOV-3-EV populations (top 20 in Supplementary Table 2), while 104 and 41 proteins were enriched exclusively when compared to Ca ion-EVs and SKOV-3-EVs, respectively (Fig. 5d). The full list of veh-EV enriched proteins (*n* = 105) was further annotated by Gene Ontology, which revealed an over-representation of biological processes such as “biological adhesion”, “cell adhesion” and “regulation of cell migration” (Fig. 5e). Collectively, these confirm that the effects of CPC-EVs on endothelial cells observed may be mediated through a collective subset of EV proteins that promote endothelial cell migration.

### Extracellular vesicle-associated PAPP-A, but not NID1, is involved in endothelial cell activation

**Fig. 6:**
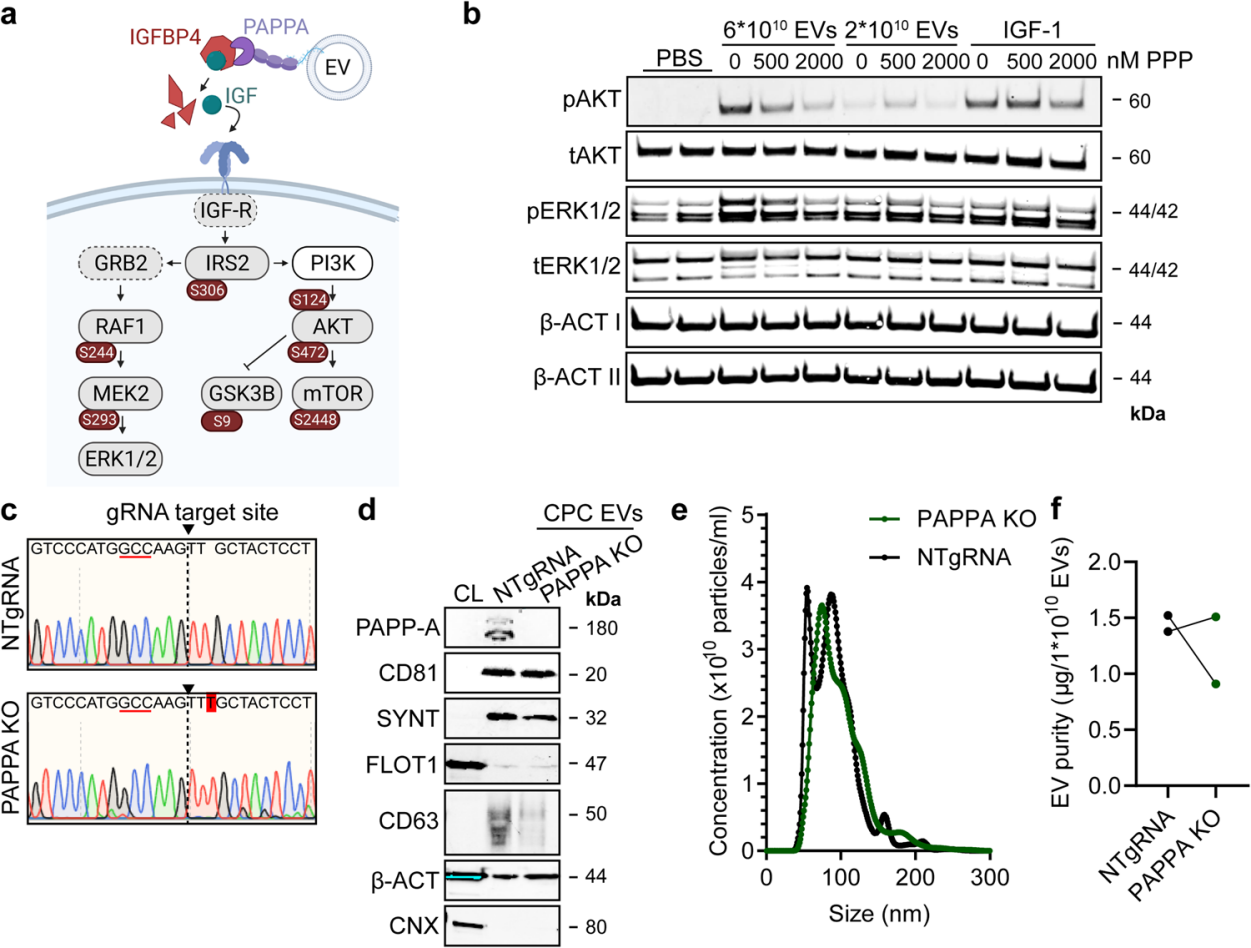
PAPPA KO-EVs were generated using CRISPR/Cas9. **a** Schematic depicting hypothesized mechanism of (intra)cellular signalling activated by EV-associated PAPP-A, based on identified proteins and significantly altered phosphosites measured in HMEC-1 upon veh-EV stimulation by (phopho)proteomic analysis. Detected proteins in HMEC-1 are displayed in grey, while significantly changing phosphosites present in cluster C1 (see Fig. 3d) are displayed in brown. **b** Representative western blot analysis of phosphorylated AKT (pAKT), total AKT (tAKT), phosphorylated ERK1/2 (pERK1/2) and total ERK1/2 (tERK1/2) in HMEC-1 treated with 6 × 10^10^ or 2 × 10^10^ CPC-EVs, or with 200 ng/mL free IGF-1 after pre-incubation with different doses of picropodophyllin (PPP). β-actin (β-ACT) was included as housekeeping protein (I = phosphorylated protein blot, II = total protein blot). Biological replicates of (**b**) are displayed in Supplementary Fig. 9g, h. **c** Sanger sequencing results confirming 1 bp insertion in exon 3 of *PAPPA* at the CRISPR/Cas9 target site of the PAPPA KO-CPC clone, compared with the NTgRNA polyclonal CPC line. **d** Western blot analysis showing the absence of PAPP-A in PAPPA KO-EVs, compared with NTgRNA-EVs; the presence of CD81, CD63, Syntenin-1 (SYNT), Flotillin (FLOT1), β-ACT, and absence of Calnexin (CNX) in both EV populations. FLOT1, β-ACT and CNX were present in CPC lysate (CL). **e** Representative NTA plot showing the size distribution and particle concentration of PAPPA KO- and NTgRNA-CPC-EVs. **f** Protein content per 1 × 10^10^ PAPPA KO- and NTgRNA-EVs of two representative experiments.

Following the more crude analysis of the CPC-EV proteome, we investigated the contribution of individual proteins to CPC-EV functionality. MS-proteomic analysis identified PAPP-A and NID1, proteins previously demonstrated to contribute to mechanisms of cardiac repair^7,27^, among the 20 most strongly enriched proteins in veh-EVs compared to Ca ion- and SKOV-3-EVs (Fig. 5b, Supplementary Fig. 4a, Supplementary Table 2). A proposed mechanism of PAPP-A function is through its expression on the EV surface through binding to glycosaminoglycans on the EV membrane (Fig. 6a)^7^. By cleavage of IGFBP4 from the IGF-1/IGFBP4 complex, it induces the release of IGF-1 in the extracellular space and subsequent activation of intracellular signalling by binding to IGF-R^28^. Phosphoproteome analysis in HMEC-1 upon veh-EV stimulation indeed revealed the specific activation of the Insulin/IGF pathway-MAPKK/MAPK cascade (Fig. 3c) and identified the phosphorylation of members of the MAPK and AKT-mTOR signalling cascades, including IRS2, RAF1, MEK2, AKT and mTOR (Fig. 6a). To study the contribution of the IGF receptor (IGF-R) to intracellular signalling upon EV stimulation, we employed the IGF-R inhibitor Picropodophyllin (PPP). PPP dose-dependently abrogated the increase in ERK1/2 and AKT phosphorylation in HMEC-1 upon CPC-EV stimulation (Fig. 6b, Supplementary Fig. 9g, h), demonstrating a contribution of the IGF-R signalling pathway in HMEC-1 activation. NID1 is a glycoprotein present in the basement membrane, reported to promote cell migration by being present on EVs derived from hepatocellular carcinoma cells^29^. Interestingly, western blotting confirmed NID1 expression in veh-EVs, but also showed the presence of a higher molecular weight NID1 form in Ca ion-EVs (Fig. 5c). This could be due to differences in NID1 glycosylation status and the bias towards detection of non-glycosylated peptides by our MS-analysis.

To investigate the influence of PAPP-A and NID1 on EV-mediated endothelial cell activation, CRISPR/Cas9 technology was employed to knock-out (KO) PAPP-A and NID1 in CPCs. Three different gRNAs targeting exon 3 and 4 of *PAPPA* and targeting the 5’UTR region and exon 1 of *NID1* were selected to identify the most appropriate target site in *PAPPA* or *NID1* for gene editing, respectively, and their genomic DNA cleavage efficiency was determined using the T7 endonuclease assay (Supplementary Figs. 4b, 5a). Based on most potent DNA cleavage efficiency, we selected gRNA 1 and 2 for PAPP-A and gRNA 3 for NID1 for the generation of KO CPC lines. Sanger sequencing revealed a mixed population of mutated alleles at the gRNA target sites (Supplementary Figs. 4c, 5b). Individual clones were generated from these CPC KO lines by clonal expansion and the presence of homozygous biallelic deletions or insertions was determined by Sanger sequencing (Supplementary Figs. 4d, 5c). One CPC clone harbouring a homozygous insertion of 1 bp in *PAPPA* and one clone harbouring a deletion of 8 bp in *NID1* were subsequently selected for the isolation of PAPP-A-depleted (PAPPA KO) and NID1-depleted (NID KO) EVs and used for further functional studies (Fig. 6c, Supplementary Fig. 5d). A polyclonal CPC line transduced with the CRISPR/Cas9 machinery, and a non-targeting gRNA (NTgRNA) served as normal CPC-EV control. PAPP-A depletion in isolated PAPPA KO-EVs was confirmed by western blotting (Fig. 6d). PAPP-A depletion resulted in reduced cell growth but did not influence cell morphology (Supplementary Fig. 4e, f) and EV production as determined by western blotting identified similar levels of CD81, Syntenin-1, Flotillin and β-actin in both the PAPPA KO- and NTgRNA-EVs (Fig. 6d). Expression of CD63 seemed slightly reduced in the PAPPA KO-EVs. Both EV populations were similar in size, as determined by NTA (Fig. 6e), and in purity (µg protein/1 × 10^10^ EVs) (Fig. 6f). Although NID1 KO-CPCs also had a slightly slower growth rate compared to NTgRNA CPCs (Supplementary Fig. 5e) and displayed a slightly more elongated morphology (Supplementary Fig. 5f), EV-release was not affected as determined by NTA and by the expression of EV markers CD81, Syntenin-1, and levels of β-actin (Supplementary Fig. 5g, h). NID1 KO-EVs contained a slightly higher protein amount per particle (µg protein/1 × 10^10^ EVs) (Supplementary Fig. 5i).

**Fig. 7:**
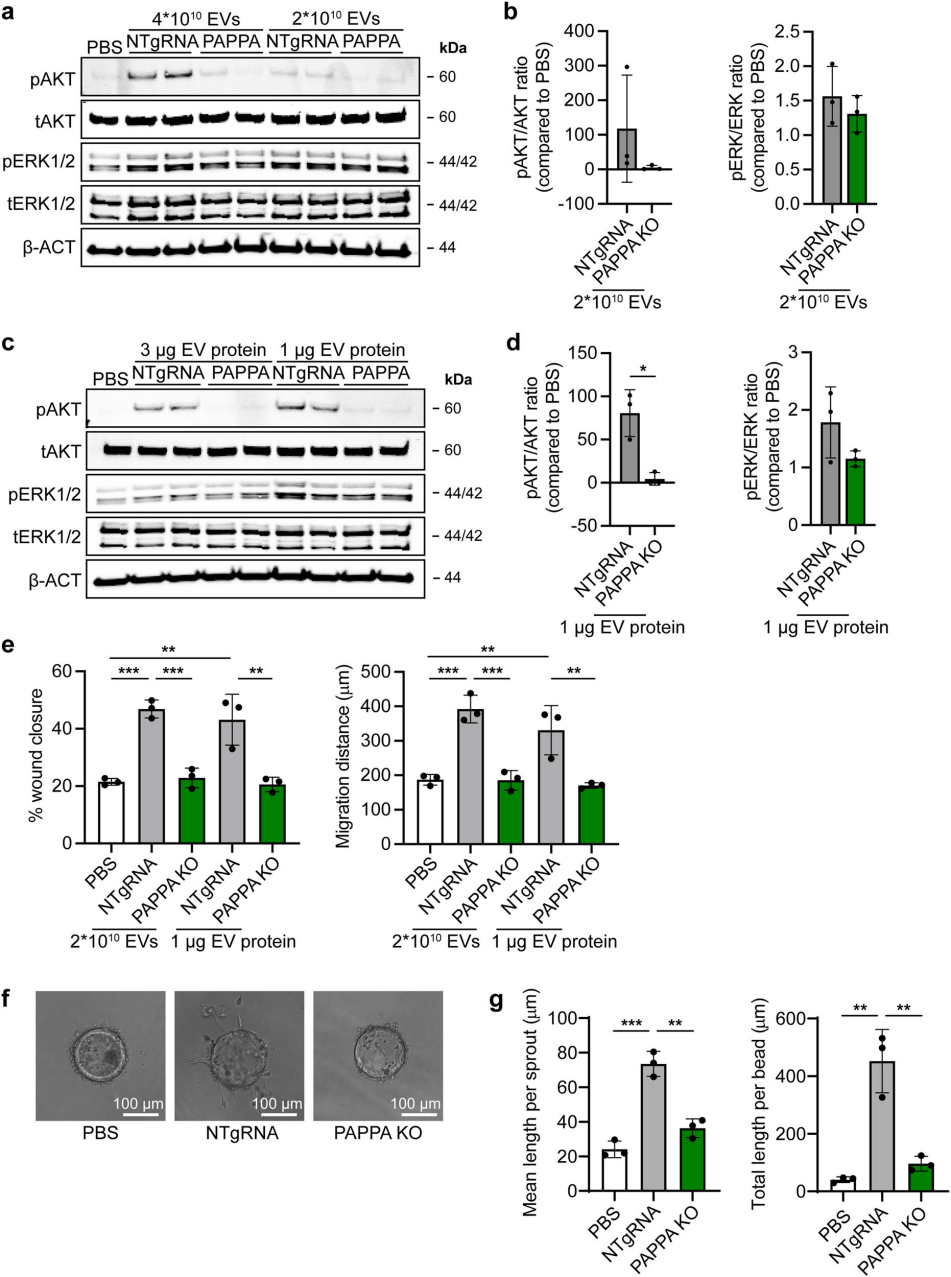
PAPPA KO-EVs showed reduced activation of intracellular signalling and activation of HMEC-1 migration and sprout formation. **a**–**d** Representative western blot analyses of pAKT, tAKT, pERK1/2 and tERK1/2 in HMEC-1 treated with PAPPA KO- and NTgRNA-EVs normalized on two doses of **a**, **b** total particle numbers or **c**, **d** total protein content. β-ACT was included as housekeeping protein. **b**, **d** Quantification of pAKT, tAKT, pERK1/2 and tERK1/2 expression levels using densitometry expressed as pAKT/AKT and pERK/ERK ratios (*n* = 3). Biological replicates of (**a**, **c**) are also displayed in Supplementary Fig. 9i–k. **e** Wound healing assay showing effects of 1 µg and 2 × 10^10^ NTgRNA- and PAPPA KO-EVs on HMEC-1 migration, analysed both as % wound closure and absolute migration distance (*n* = 3, technical replicates. Data are representative of three biologically independent experiments). **f**, **g** Sprouting assay showing NTgRNA- and PAPPA KO-EV-induced HMEC-1 sprout formation on beads, analysed both as (**g**) mean length per sprout and total sprout length per bead (*n* = 3, technical replicates. Data are representative of two biologically independent experiments). Data are presented as mean ± SD. *p < 0.033, **p < 0.0021, ***p < 0.0002.

Next, we evaluated the influence of PAPP-A and NID1 KO on EV-induced angiogenesis. Compared to NTgRNA-EVs, PAPPA KO-EVs were less bioactive in inducing AKT- phosphorylation in HMEC-1, when EV addition was normalized on total particle number or total protein content (Fig. 7a–d, Supplementary Fig. 9i–k). Moreover, a trend towards reduced ERK1/2 phosphorylation was observed. NID1 KO did not affect EV-stimulating activity (Supplementary Fig. 6a–d). In an HMEC-1 scratch assay, PAPPA KO-EVs were also less potent in inducing wound closure, calculated both as percentage wound closure as absolute migration distance, when normalized for total particle number or total protein content (Fig. 7e). Moreover, PAPPA KO-EVs lost part of their ability to induce the formation of sprouts in an in vitro sprouting assay (Fig. 7f, g). In contrast, there was no difference in activity between NID1 KO- and NTgRNA-EVs in inducing wound closure (Supplementary Fig. 6e). NID1 has been hypothesized to be involved in cardiac repair by binding to the αVβ integrin receptor and thereby activating downstream MAPK signalling^27^. However, EV-induced AKT and ERK1/2 phosphorylation and wound closure was not influenced by the pre-treatment of an IntegrinαVβ3 antibody (Supplementary Fig. 7a–d). In addition, recombinant NID1 did not induce AKT and ERK1/2 phosphorylation nor was able to induce wound closure in the HMEC-1 scratch assay. This implies that EV-associated NID1 is not contributing to CPC-EV functionality in our in vitro assays. Overall, our data suggest that PAPP-A is enriched on CPC-EVs and is involved in EV-mediated endothelial cell activation via the IGF-R signalling pathway.

### Both EV-associated and co-isolated proteins are contributors to CPC-EV-mediated cell activation

**Fig. 8:**
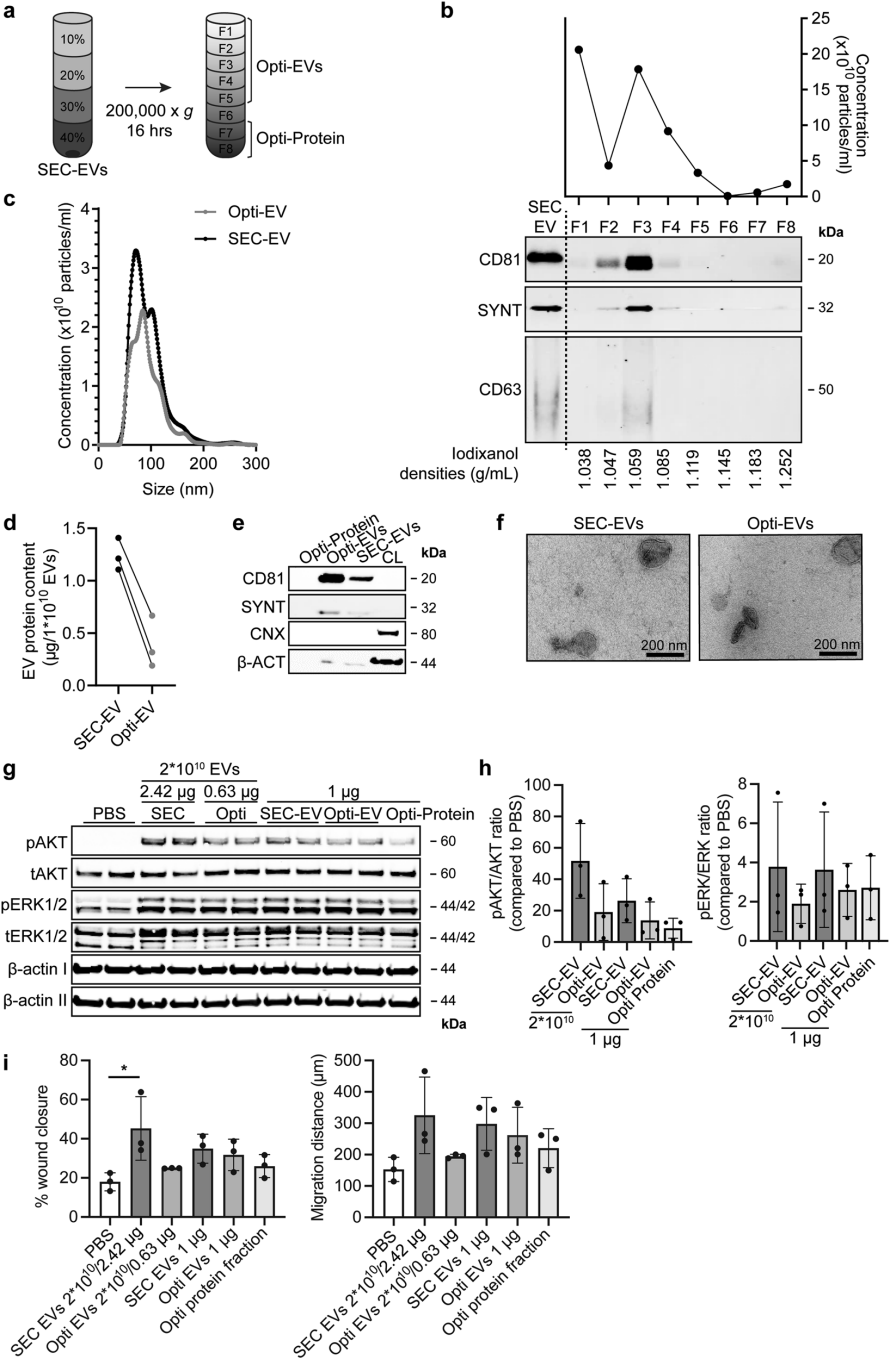
Both co-isolated proteins and EV-associated factors contribute to HMEC-1 activation. **a** Schematic overview of the EV isolation protocol to obtain pure Opti-EVs. SEC-EVs were loaded in the bottom of a discontinuous Optiprep^TM^ gradient and ultracentrifuged for 16 h. **b** Resulting fractions (F1–8) were analysed for particle number by NTA (upper panel) and the presence of EV-marker proteins CD81, CD63 and Syntenin-1 (SYNT) by western blotting. Equal volumes of each sample were analysed. Iodixanol concentration was measured in each fraction (lower panel). Fractions 1–5 were pooled and concentrated (Opti-EVs). **c** Representative NTA plot showing the size distribution and particle concentration of SEC- and purified Opti-EVs. **d** Protein content per 1 × 10^10^ SEC- and Opti-EVs of three representative experiments. **e** Western blot analysis showing presence of CD81, SYNT, β-actin (β-ACT) in purified Opti-EV- and protein fractions, compared with crude SEC-EVs. Calnexin (CNX) was only present in CPC lysate (CL). Complete β-ACT blot is displayed in Supplementary Fig. 10. **f** Representative TEM image of SEC- and Opti-EVs. **g**, **h** Representative western blot analysis of phosphorylated AKT (pAKT), total AKT (tAKT), phosphorylated ERK1/2 (pERK1/2) and total ERK1/2 (tERK1/2) in HMEC-1 treated with SEC-EVs and Opti-EVs normalized on total particle numbers, and with SEC-EVs, Opti-EVs and Opti-protein fraction normalized on total protein content. β-ACT was included as housekeeping protein (I = phosphorylated protein blot, II = total protein blot). **h** Quantification of pAKT, tAKT, pERK1/2 and tERK1/2 expression levels using densitometry expressed as pAKT/AKT and pERK/ERK ratios (*n* = 3). Biological replicates of (**g**) are displayed in Supplementary Fig. 9e, f. **i** Wound healing experiment showing effects SEC- and Opti-EVs on HMEC-1 migration normalized both on total particle number and total protein content, analysed both as % wound closure and absolute migration distance. Addition of Opti-protein fraction was normalized on volume (*n* = 3, technical replicates. Data are representative of three biologically independent experiments). Data are presented as mean ± SD. *p < 0.033.

SEC is a widely adopted method of EV isolation but does not yield a completely pure EV population, as also a small fraction of soluble proteins may be co-isolated. As a potential contribution of these co-isolated proteins to EV function has been postulated, we set out to investigate the contribution of co-isolated proteins to the functionality of SEC isolated CPC-EV preparations. We indeed confirmed the enrichment of ECM proteins in veh-EVs (Supplementary Table 2) and we attempted to dissect if these proteins were packaged in EVs or co-isolated in our preparations. To this end, Optiprep^TM^ density gradient centrifugation after SEC was employed to further separate EVs from co-isolated proteins (Fig. 8a). Western blot analysis of the different fractions confirmed the presence of EV-markers CD81, Syntenin-1 and CD63 mainly in fractions 2–4 (Fig. 8b). Total particle numbers in each fraction were measured using NTA and showed presence of particles in fractions 2–4 although particles were also present in the top fraction (F1) (Fig. 8b). EV-containing fractions 1–5 (Opti-EVs) and co-isolated protein-containing fractions 7 and 8 (Opti-protein), as showed by silver stain (Supplementary Fig. 3a), were collected and concentrated using 100- or 10 kDa cut-off spin filters. Opti-EVs and SEC-EVs showed similar size distribution (Fig. 8c), while Opti-EVs contained less protein content per particle compared with SEC-EVs, suggesting higher purity as expected (Fig. 8d). Western blotting demonstrated an increased relative expression of CD81 and Syntenin-1, and absence of Calnexin in Opti-EVs compared with SEC-EVs, again confirming higher purity (Fig. 8e*)*. TEM confirmed the presence of membrane enclosed particles in both EV preparations (Fig. 8f). As we found that iodixanol increased EVs’ bioactivity to induce AKT- and ERK1/2 phosphorylation and wound closure (Supplementary Fig. 3b, c), iodixanol concentrations in all samples were kept constant (2%) in all functional assays to exclude the direct functional effect of iodixanol. In an endothelial activation assay, Opti-EVs induced AKT and ERK1/2 phosphorylation, although to a lesser extent than SEC-EVs (Fig. 8g, h, Supplementary Fig. 9e, f). The Opti-protein fraction also demonstrated limited HMEC-1 stimulating capacity. In a HMEC-1 scratch assay, both Opti-EVs and the Opti-protein fraction displayed some but reduced migration-stimulating capacities compared with SEC-EVs (Fig. 8i). To investigate the association of PAPP-A to CPC-EVs, we investigated the expression of PAPP-A across the different fractions after iodixanol gradient isolation. Western blotting demonstrated that PAPP-A was present both in tetraspanin-positive and negative fractions 4–7 (Supplementary Fig. 8). Together, these results suggest that both EV-associated and co-isolated proteins present in the crude SEC-EV preparations can contribute to endothelial cell activation.

## Discussion

CPC-EV administration has been shown to improve cardiac function by promoting different cellular processes including angiogenesis in several preclinical animal models^1^. In line with previous studies, we confirmed that CPC-EVs are active inducers of endothelial cell activation and functionality^5,6^. Despite promising preclinical results of EV therapeutics for tissue regeneration, translation into the clinics is still hampered by in vivo reproducibility due to EV heterogeneity and poor understanding of functional mediators in EVs^10,11^. In order to advance CPC-EVs as therapeutics, more insights into functional EV cargo and mechanisms of EV-mediated cell activation are required. In this work, we investigated the pro-angiogenic composition of CPC-EVs.

To increase EV release by CPCs we explored the use of calcium ionophore A23187, a compound described to stimulate the release of EVs through the increase of intracellular Ca^2+^ levels^23–26^. Interestingly, CPC exposure to calcium ionophore did not increase EV release, but did result in a less functional EV population as shown in different HMEC-1 angiogenesis assays. We employed the differences in functionality between CPC veh- and Ca ion-EVs to investigate the specific activation of intracellular signalling by employing phosphoproteomic analysis of HMEC-1 upon stimulation with both EV populations. This is one of the first studies investigating the activation of intracellular signalling in recipient cells by means of phosphoproteomics upon EV stimulation, which could identify the increased phosphorylation of members of the P13K-AKT and (Insulin/IGF-) MAPK signalling pathways. We observed significant changes at the phosphoproteome level but not at the proteome level within 30 min after HMEC-1 stimulation. In addition, we demonstrated that the ability of EVs to activate intracellular signalling pathways was lost after the removal of EV-surface proteins using proteinase K, as previously shown for EVs derived from other sources^30^. This hints toward fast signal transduction via receptor-ligand interactions instead of EV-content delivery through EV internalization. Indeed, recent studies demonstrate that EVs contain only a very limited number of specific miRNAs^31–35^, corroborating the importance of investigating the contribution of other EV content such as proteins to EV functionality.

Several groups have previously characterized the proteomic composition of stem- and progenitor cell-derived EVs, which is currently available via several databases^36–38^. Identification of functional components, however, was done mainly by comparisons of EVs derived from different cell types^7,39^ or after differences upon cell preconditioning^40,41^. Here, we compared the proteomic composition of functionally distinct EV types, released from the same cell type and donor, thereby eliminating potential differences in content caused by differences in isolation technique and biological variability of cell type and donor. MS profiling identified a multitude of proteins differentially expressed in veh-EVs relative to Ca ion-EVs. These veh-EV enriched proteins were over-represented in PANTHER gene ontology biological process ‘regulation of cell migration’ (top 3), which demonstrated our approach as a method for unbiased identification of proteins potentially involved in EV-induced cell migration.

MS-proteomic analysis identified candidate proteins PAPP-A and NID1 enriched in CPC-EVs. Employing CRISPR/Cas9, we set out to identify their contribution to EV function. KO of NID1 did not influence HMEC-1 stimulation capacities of CPC-EVs. In contrast, depletion of PAPP-A from CPC-EVs yielded EVs with reduced capacity to induce in vitro angiogenesis. The presence of PAPP-A on CPC-EVs and its involvement in cardioprotection was previously demonstrated by Barile et al.^7^. PAPP-A was present on the surface of CPC-derived EVs and abrogated EV-mediated protection against staurosporine-induced cell death in HL-1 cells. As loss in protection was evoked only after addition of the IGF-1/IGFBP4 complex, where EV-bound PAPP-A is suggested to cleave the IGF-1/IGFBP4 complex and thereby leads to IGF-1 release and subsequent target cell activation^28^. Based on measured changes at the phosphoproteome level, we could identify the Insulin/IGF-MAPK pathway as most enriched pathway, providing another link between PAPP-A and the activation of IGF-1-MAPK signalling. This is further supported by our observation that the IGF-R inhibitor PPP abrogated CPC-EV-induced HMEC-1 activation.

EVs released from different stem cell types were reported to have similar pro-regenerative properties in vitro and in vivo^42^, but also differences in functionality have been discovered between different organs and disease states, as reflected by differences in identified functional EV cargo^9,10^. EVs derived from different (stem) cell donors might be enriched in different functional proteomic cargo, as the presence of both PAPP-A and NID1 in EVs has been reported before^38,43^, but not reproducibly^44^. The proteomic profiles of different stem cell-derived EVs might largely differ due to EV heterogeneity caused by different methods used for EV isolation, as for example SEC is likely to better remove overabundant soluble proteins compared with other techniques^13^, but also by the variability between cell donors and culture state^2^. Here, we investigated the contribution of individual CPC-EV-associated proteins to stimulate pro-angiogenic processes in vitro, which clearly contribute but might not explain all pro-regenerative properties of (CPC-)EVs in vivo. Whether PAPP-A contributes to EV-regenerative properties in other contexts remains to be investigated.

Although initial studies identified EVs as functional components of the stem cell secretome, more recent studies have hypothesized that also soluble co-isolated factors contribute to observed EV-mediated therapeutic effects, which might even be synergistic^14–18^. These observations suggest that released therapeutic stimuli are likely not to be mediated exclusively by stem cell-derived EVs. We observed that many of identified proteins enriched in veh-EVs were known components of the ECM. This raised the question whether these proteins were truly associated to EVs or just present as a result of co-isolation. Although other studies have attributed EV-mediated function to members of the ECM by being present in EV preparations^7,29,44^, the influence of co-isolated proteins in functional CPC-EV preparations, isolated by SEC, has not been investigated yet. Here, we show that despite SEC isolation that separates CPC-EVs from proteins based on size differences, the EV preparation still contains co-isolated proteins or other factors that contribute to EV-function. Indeed, both co-isolated factors and the further purified EVs upon iodixanol density gradient centrifugation displayed some pro-angiogenic properties, as also demonstrated by others^45–49^. We hypothesize a contribution of both EV-associated factors and co-isolated proteins to CPC-EV function, which might potentially be synergistic. To get more insights into the presence of PAPP-A in our CPC-EV preparations, we investigated the presence of PAPP-A in the different fractions after iodixanol gradient separation. We observed its presence both in fractions in which also tetraspanins were detected, but also in higher-density fractions, which implies that PAPP-A might be associated to tetraspanin-positive EVs, but also on tetraspanin-negative EVs. Alternatively, the glycosaminoglycan bonds by which PAPP-A is attached to the EV membrane may get disrupted during the iodixanol gradient density centrifugation, leading to the removal of PAPP-A from the EV protein corona during the EV isolation process. However, further research should be performed to determine the composition and contribution of a protein corona to CPC-EV function, including the presence of specific candidate proteins such as PAPP-A.

In conclusion, we demonstrate that CPC-EVs can promote endothelial cell activation and cellular migration and identified the proteomic composition of functional CPC-EVs that potentially contribute to these processes. Furthermore, we characterized downstream signalling upon CPC-EV stimulation in endothelial cells. We demonstrated that both EV-associated factors and co-isolated proteins in the CPC-EV preparation contributed to endothelial cell activation. Using CRISPR/Cas9 to knock-out individual proteins from EVs, we identified PAPP-A but not NID1 to contribute to CPC-EV function. These results support the idea that specific proteins present in SEC-EV preparations can selectively induce specific signals in recipient cells to regulate processes such as angiogenesis. This knowledge of the pro-angiogenic components and localization of these components in CPC-EV preparations can be further applied in future studies exploring the use of EVs for therapeutic application.

## Methods

### Cell culture and EV isolation

Cardiac progenitor cells (CPCs, donor HFH070809) were obtained from human fetal hearts as described before^50^. Human fetal heart tissue was obtained by individual permission using standard written informed consent and after approval of the ethics committee of Leiden University Medical Center, The Netherlands. This is according to the principles outlined in the Declaration of Helsinki for the use of human subjects or tissue. Primary CPCs (passage 9–17) were cultured in SP++ medium (66% M199 medium (Gibco), 22% EGM-2 (Lonza), 10% fetal bovine serum (FBS) (Life-Tech), 1% Penicillin/Streptomycin (P/S) (Invitrogen), and 1% MEM nonessential amino acids (Gibco)^12^. Human microvascular endothelial cells (HMEC-1, ATCC) were cultured in MCDB131 medium (Invitrogen) supplemented with 10% FBS, 1% P/S, 5% L-glutamine (Invitrogen), 50 nM Hydrocortisone (Sigma) and 10 ng/ml rhEGF-1 (Peprotech/Invitrogen)^51,52^. CPCs and HMEC-1 were cultured in flasks coated with 0.1% gelatin. Epithelial SKOV-3 ovarian adenocarcinoma cells (ATCC) and HEK293fT cells were cultured in DMEM (Gibco) supplemented with 10% FBS and 1% P/S. All cells were cultured at 5% CO_2_ at 37 °C and passaged at 80–90% confluency after digestion with 0.25% trypsin. CPC-EV-conditioned medium was prepared by culturing CPCs for 3 days on SP++ medium until 80% confluency was reached, followed by 24 h of culture on basal FBS- and supplement-free M199 medium containing 1 µM calcium ionophore A23187 (Sigma) or vehicle (0.0125% DMSO). For direct comparative experiments, (knock-out) CPC clonal lines of the same passage were used. SKOV-EV-conditioned medium was obtained by culturing of SKOV-3 until 80% confluency, followed by medium replacement to DMEM without any additives for 24 h.

### EV isolation by ultrafiltration and size-exclusion chromatography (SEC)

Conditioned medium was collected after 24 h, centrifuged at 2000 × *g* for 15 min and 0.45 µm filtered (0.45 µm aPES bottle top Nalgene filter) to remove cellular debris. Conditioned medium was concentrated using 100-kDA molecular weight cut-off (MWCO) Amicon Ultra-15 spin filters (Merck Millipore) or by Tangential Flow Filtration (TFF) using a Minimate TFF capsule with 100-kDa MWCO, and subsequently loaded onto a S400 high-prep column (GE Healthcare) using an ÄKTA start system (GE Healthcare) containing an UV 280 nm flow cell. Fractions containing EVs were pooled, filtered using a 0.45 µm surfactant-free cellulose acetate membrane (SCFA) syringe filter (Corning) and again concentrated using a 100-kDa MWCO Amicon Ultra-15 spin filter (Merck Millipore). EVs were stored at 4 °C for a maximum of 2 days until further use.

### EV purification by Optiprep^TM^ density gradient

To obtain more pure EVs, SEC-EVs were further purified employing iodixanol density gradient ultracentrifugation. Solutions of 5%, 10%, 20 and 40% iodixanol were made by mixing PBS with OptiPrep^TM^ 60% (w/v) aqueous iodixanol solution. A discontinuous gradient was prepared by layering 3 mL of each solution on top of each other in a 14.5 mL open top polyallomer tube (Beckman Coulter). EVs were loaded in the 40% solution at the bottom. Tubes were centrifuged at 100,000 × *g* and 4 ^°^C for 16 h (SW 32.1 Ti rotor, Beckamn Coulter). From the top to the bottom, 8 fractions of 1.5 mL each were collected. Each fraction was weight scaled to determine the density. Particle concentration was determined using nanoparticle tracking analysis (NTA). EV-containing fractions (fractions 1–5) were pooled, diluted with PBS, and concentrated using 100-kDa MWCO Amicon Ultra-15 or Ultra-4 spin filters (Merck Millipore). Protein-containing fractions (fractions 7 and 8) were pooled, diluted with PBS, and concentrated using 10-kDa MWCO Amicon Ultra-15 spin filters (Merck Millipore). Remaining iodixanol was removed by washing with >20 mL of PBS, and complete removal was confirmed using an optical eclipse brix handheld refractometer (Bellingham+Stanley).

### Proteinase K treatment

EVs were incubated in a final concentration of 100 µg/mL Proteinase K (Promega) for 30 min at 37 °C. Proteinase K was inactivated by diluting the EV sample to 1 mL in PBS supplemented with protease inhibitor (Roche), and EVs were subsequently separated from fractionated proteins by SEC using a Sepharose CL-4B column connected to an ÄKTA start system (GE Healthcare) containing an UV 280 nm flow cell. An EV sample without treatment with Proteinase K, but with the subsequent isolation and concentrating steps, was taken along in parallel and served as untreated control. EV-containing fractions were pooled and again concentrated using a 100-kDa MWCO Amicon Ultra-4 spin filter (Merck Millipore).

### Nanoparticle tracking analysis and zeta potential measurements

NTA was performed using a Nanosight NS500 system (Malvern Technologies). EVs were diluted in PBS and three videos of 30 s were captured using a camera level of 15 and a detection threshold of 5. Size and particle concentration was determined with the Nanosight NTA 3.3 software (Malvern Technologies). Particle surface potential was measured by laser Doppler electrophoresis on a Zetasizer Nano Z (Malvern Panalytical, Malvern, UK). Samples were diluted in 0.1× DPBS and sample was measured for 20 runs in triplicate.

### EV protein determination and western blot

Total protein concentrations of EV samples were determined using the Pierce microBCA Protein Assay Kit (Thermo Fisher Scientific) according to manufacturer’s protocol after lysis in 1x RIPA buffer (Abcam). EV proteins were analysed with western blot. Samples were mixed with NuPAGE sample buffer (Thermo Fisher Scientific) and NuPAGE sample reducing agent (Thermo Fisher Scientific) and heated to 90 °C for 10 min to reduce proteins. Equal protein amounts were loaded on a 4–12% Bis-Tris polyacrylamide gel (Thermo Fisher Scientific) and subjected to electrophoresis. Proteins were blotted on a Immobilon-FL polyvinylidene difluoride (PVDF) membranes (Merck Millipore), which were subsequently blocked with 50% v/v Odyssey Blocking Buffer (LI-COR Biosciences) in Tris-buffered saline (TBS). Antibodies were incubated in 50% v/v Odyssey Blocking Buffer in TBS containing 0.1% v/v Tween 20 (TBS-T). Primary antibodies used were mouse anti-CD63 (Abcam, 1:1000), rabbit anti-CD9 (Abcam, 1:1000), mouse anti-ALIX (Thermo Scientific, MA1-83977), rabbit anti-Calnexin (GeneTex, GTX 101676, 1:1000), mouse anti-β-actin (Sigma, 1:5000), rabbit anti-TSG101 (Abcam, 1:1000), mouse anti-Syntenin-1 (Origene, TA504796, 1:1000), rabbit anti-Annexin A1 (Abcam, ab214486, 1:1000), goat anti-Nidogen-1 (R&D Systems, AF2570), goat anti-PAPP-A (R&D Systems, AF2487), mouse anti-PAPP-A (Hytest, 4PD4) and mouse anti-CD81 (clone B-11, Santa Cruz, 1:1000). Secondary antibodies included Alexa680-conjugated goat anti-mouse (Thermo Fisher Scientific, 1:7500) and IRG800-conjugated goat anti-rabbit (LI-COR Biosciences, 1:7500). Imaging was performed on an Odyssey Infrared Imager (LI-COR Biosciences) at 700 and 800 nm. CD63 was detected in non-reduced protein samples.

### HMEC-1 stimulation

140,000 HMEC-1 were plated in a 48-wells plate one day before stimulation. 3 h before stimulation, HMEC-1 were starved using basal MCDB131 medium. 6 × 10^10^, 2 × 10^10^, 3 µg or 1 µg of EVs were added in duplicate for 30 min to stimulate HMEC-1. PBS was supplemented as negative control. For inhibition experiments, different doses (100–2000 nM) of picropodophyllin (PPP; Calbiochem) were added 3 h, or 10 µg/mL mouse anti-IntegrinαVβ3 antibody (Novus Biologicals) was added 1 hr before EV addition into the basal medium. Stimulation with 200 ng/mL recombinant IGF-1 (PromoCell) or 1 µg/mL Nidogen-1 (R&D Systems) was included as positive control. For phophoproteomic analysis, 260,000 HMEC-1 were plated in a 24-wells plate one day before stimulation with 10 × 10^10^ EVs or PBS. Cells were lysed using cOmplete Lysis-M EDTA-free lysis buffer (Roche) supplemented with PhosSTOP phosphatase inhibitors (Roche) and centrifuged at 14,000 × *g* for 10 min at 4 °C to remove cellular debris. Supernatants were used for further western blotting experiments and (phospho)proteomic analysis as described below.

### Western blot for pAKT and pERK1/2 expression

Total protein content of HMEC-1 lysates was determined using a Pierce microBCA Protein Assay Kit (Thermo Fisher Scientific) according to manufacturer’s instructions, and proteins were analysed with western blot. Samples were mixed with NuPAGE sample reducing agent (Thermo Fisher Scientific) and NuPAGE sample buffer (Thermo Fisher Scientific), heated to 90 °C for 10 min, and subjected to electrophoresis over 4–12% Bis-Tris polyacrylamide gels (Thermo Fisher Scientific). Proteins were blotted on Invitrolon PVDF membranes (Thermo Fisher Scientific) using the iBlot 2 Dry blotting system (Thermo Fisher Scientific). Membranes were subsequently blocked with 5% Bovine serum albumin (BSA) or with 50% v/v Intercept Blocking Buffer (LI-COR Biosciences) in Tris-buffered saline (TBS). Primary and secondary antibody incubations were performed in 0.5% BSA in TBS containing 0.1% Tween 20 (TBS-T) or in 50% v/v Intercept Blocking Buffer in TBS-T. Primary antibodies included rabbit anti-phospho-ERK (Phospho-p44/p42 MAPK (Erk1/2) (Thr202/Tyr204, Cell Signaling Technology, 1:1000), rabbit anti-ERK (p44/p42 (Erk1/2), Cell Signaling Technology, 1:1000), rabbit anti-phospho-AKT (Ser473, Clone D9E, Cell Signaling Technology, 1:1000), rabbit anti-AKT (Cell Signaling Technology, 1:1000) and mouse anti-β-actin (Sigma, 1:5000). Secondary antibodies included HRP-conjugated goat anti-rabbit antibody (DAKO) or Alexa680-conjugated goat anti-mouse (Thermo Fisher Scientific, 1:7500) and IRG800-conjugated goat anti-rabbit (LI-COR Biosciences, 1:7500). Proteins were detected with chemiluminescent peroxidase substrate (Sigma) using a Chemi DocTM XRS+ system (Bio-Rad) and Image LabTM software, or imaging was performed on an Odyssey Infrared Imager (LI-COR Biosicences) at 700 nm and 800 nm.

### Endothelial wound closure assay

A scratch was made in a monolayer of HMEC-1 in a 48-wells plate and any floating cells were removed. Medium was replaced to basal MCDB131 with or without 2 × 10^10^ or 1 µg EVs or equal volumes of PBS. MCDB131 medium supplemented with 20% FBS was included as positive control. For inhibition experiments, 10 µg/mL mouse anti-IntegrinαVβ3 antibody (Novus Biologicals) was added simultaneously with EV addition. Stimulation with 1 µg/mL Nidogen-1 (R&D Systems) was included as positive control. At 0 and 6 h, bright field pictures were taken using an EVOS microscope (Life Technologies). Percentage closure and absolute migration distance was determined after 6 h. Relative wound closure was calculated relative to the PBS control.

### Sprout formation assay

Two million HMEC-1 were incubated in suspension with Cytodex 3 microcarrier beads (Cytiva) for 4 h at 37 °C under regular agitation to enable cell attachment to the beads. Afterwards, beads with cells were incubated at 37 °C overnight. The following day, a mixture of basal MCDB131 medium with 4 µg EV treatments or equal volumes of PBS and growth factor reduced Matrigel (Corning, ratio 4:1:1) was prepared and ~50 beads were embedded in between two layers of the mixture in each well of a 96-wells-plate. After solidification of the Matrigel mixture, 200 μl of basal MCDB131 medium was added on top and plates were incubated at 37 °C. After 72 h, bright field images of 6 beads per well were taken using an EVOS microscope (Life Technologies). Images were analysed for number of sprouts, mean length per sprout and total length per bead using ImageJ with the NeuronJ plugin.

### CRISPR/Cas9 plasmid construction and stable CPC line generation

Single guide RNAs (sgRNAs) specific for human PAPP-A and NID1 were designed using the CRISPOR design tool (http://crispor.tefor.net/) and were screened for the Homo Sapiens genome to minimize potential off-target effects. All sgRNA sequences and PCR primers are listed in Supplementary Table 1. sgRNA expressing plasmids were achieved by cloning of the sgRNA in the LentiCRISPv2 vector (Addgene Plasmid number 52961) by oligo annealing of phosphorylated sgRNA oligo duplexes into Esp3I sites. Phosphorylated sgRNA oligo duplexes were formed by ligating top and bottom strand oligos (HPLC-purified, IDT) with T4 PNK ligase at 37 °C for 30 min, followed by 5 min incubation at 95 °C and a ramp-down of 5 °C per min to 25 °C. To generate polyclonal stable CPC lines, CPCs were infected with CRISPv2-expressing lentiviruses. HEK293fT cells were plated in 6-well plates and transfections of plasmid DNA were performed using Lipofectamine 3000 reagent (Life Technologies) according to the manufacturer’s instructions when cells were at 50–60% confluency. 1 µg LentiCRISPv2 plasmid (Addgene #52961) was mixed with 1 µg pCMV delta R8.2 (Addgene#12263) and 0.5 µg pCMV-VSV-G (Addgene #8454) helper plasmids and complexed in a 1 µg to 1 µl ratio with Lipofectamine 3000 reagent and in a 1 µg to 2 µl ratio with P3000 reagent. The complexes were added to the cells and incubated for 48 h before harvesting of the virus-containing medium. Cell debris was removed by centrifugation at 350 × *g* for 15 min and filtration using a 0.45 µm SCFA syringe filter (Corning). CPCs were cultured in a 24-wells plate until 50% confluency before supplementation with 1 mL of virus-containing medium containing 8 ng/mL polybrene. After 24 h, medium was changed to SP + + medium and stable polyclonal cell lines were generated by puromycin selection starting 48 h after transduction. Individual knock-out (KO) clones were obtained by serial diluting polyclonal KO lines to single-cell suspensions and expanding single cells in a 96-wells plate. Once confluent, genomic DNA was extracted using the GeneJet Genomic DNA Purification kit (Thermo Scientific) following the recommended protocol and investigated for a homozygous biallelic mutation using Sanger sequencing.

### Genomic DNA extraction and T7 endonuclease assay

Genomic DNA was extracted from stable polyclonal CPCs using the GeneJet Genomic DNA Purification kit (Thermo Scientific) following the recommended protocol. The genomic region flanking the CRISPR/Cas9 target site was first amplified by PCR using Q5 Hot Start High-Fidelity 2x Master Mix (NEB) on 100 ng DNA template. After an initial incubation at 98 °C for 30 s, 35 cycles of 98 °C for 10 s, 65 °C for 30 s, and 72 °C for 45 s were used followed by a final extension at 72 °C for 10 min. Afterwards, amplicons were subjected to a melting and re-annealing process in NEBuffer 2 (New England Biolabs) to allow heteroduplex formation: 95 °C for 5 min, 95 °C to 85 °C ramping at 2 °C/s, 85 °C to 25 °C at 0.1 °C/s. After re-annealing, products were treated with T7 Endonuclease I (New England Biolabs) for 30 min at 37 °C following the manufacturer’s recommended protocol and the digestion products were separated on 1% agarose gel containing ethidium bromide. Gels were imaged with a Gel Doc XR+ imaging system (Bio-Rad). To confirm the frameshift at the genomic DNA level at the CRISPR/Cas9 target site, amplified PCR products were analysed by Sanger sequencing.

### EV lysis and digestion for proteomic analysis

Biological triplicates of isolated EVs were stored at −80 °C and protein composition was analysed by mass spectrometry (MS). For this, EVs were lysed by 2% SDS lysis buffer (2% SDS, 50 mM HEPES pH 7.6, 1 mM DTT) and prepared for MS analysis using a modified version of the SP3 protein clean up and digestion protocol^53^. All extracted protein from each sample was alkylated with 4 mM Chloroacetamide. Sera‐Mag SP3 bead mix (20 µl, Cytiva) was transferred into the protein sample together with 100% Acetonitrile to a final concentration of 70%. The mix was incubated under rotation at room temperature (RT) for 18 min. The mix was placed on the magnetic rack and the supernatant was discarded, followed by two washes with 70% ethanol and one with 100% acetonitrile. The beads-protein mixture was reconstituted in 100 µl LysC buffer (0.5 M Urea, 50 mM HEPES pH: 7.6 and 1:50 enzyme (LysC) to protein ratio) and incubated overnight. Finally, trypsin was added in 1:50 enzyme to protein ratio in 100 µl 50 mM HEPES pH 7.6 and incubated overnight followed by SP3 peptide clean up. Briefly, 20 µl Sera‐Mag SP3 bead mix (10 µg/µl) was added to the sample. Next, 100% acetonitrile was added to achieve a final concentration of 95%. Samples were pipette-mixed and incubated for 20 min at RT and then placed on a magnetic rack. The supernatant was aspirated, discarded and the beads were washed in 180 µl of acetonitrile. Samples were removed from the magnetic rack and beads were reconstituted in 20 µl of (3% Acetonitrile, 0.1% formic acid) solution, followed by 1 min of sonication. Then the beads were placed on a magnetic rack again and the supernatant was recovered and transferred to a MS-vial. The peptides were dissolved in LC mobile phase A (3% acetonitrile (ACN), 0.01% FA) and were injected in the liquid chromatography (LC)-MS/MS system.

### LC–MS/MS analysis of EVs

Data were acquired using a Dionex UltiMate™ 3000 RSLCnano System coupled to a Q-Exactive mass spectrometer (Thermo Scientific). Samples were trapped on a C18 guard desalting column (Acclaim PepMap 100, 75 µm × 2 cm, nanoViper, C18, 5 µm, 100 Å), and separated on a 50 cm long C18 column (Easy spray PepMap RSLC, C18, 2 µm, 100 Å, 75 µm × 50 cm). The nano capillary solvent A was 95% water, 5% DMSO, 0.1% formic acid; and solvent B was 5% water, 5% DMSO, 95% acetonitrile, 0.1% formic acid. At a constant flow of 0.25 μl/min, the curved gradient went from 6% B up to 43% B in 180 min, followed by a steep increase to 100% B in 5 min.

The mass spectrometer was operated in data-dependent mode. Peptides were ionized in a nESI source at 1.9 kV and focused at 60% amplitude of the RF lens. Full scan MS1 spectra from 400 to 1200 *m*/*z* were acquired in the Orbitrap at a resolution of 60,000 with the AGC target set to 1 × 10^6^ or for a maximum injection time of 250 ms. For each cycle, the top 10 most abundant precursor ions were isolated (with an isolation window of 2 *m*/*z*) for fragmentation while precursor ions with charge states 1 or unassigned were excluded for fragmentation. Dynamic exclusion was set to a duration of 20 s. Fragmentation was done using fixed HCD normalized collision energy of 30%. Fragment ions were accumulated until a target value of 2 × 10^5^ ions was reached or for a maximum injection time of 140 ms before injection in the Orbitrap for MS2 analysis at a resolution of 30,000.

### HMEC-1 protein digestion for (phospho)proteome analysis

Total protein content of stimulated HMEC-1 lysates was determined using a microBCA Protein Assay Kit (Thermo Fisher Scientific) according to manufacturer’s instructions. Two biological replicates were pooled to obtain 50 µg protein for subsequent phospho-enrichment analysis. Proteins were precipitated by the methanol/chloroform method: 1 volume of sample was sequentially mixed with 4 volumes of methanol (Sigma-Aldrich), 1 volume of chloroform (Sigma-Aldrich) and 3 volumes of water. The mixture was centrifuged at 5000 rpm for 10 min at RT and the upper layer was removed. Then, 3 volumes of methanol were incorporated and centrifuged at 5000 rpm for 10 min at RT, and the liquid phase was discarded while the pellet (proteins) was allowed to air dry. The protein pellets were reconstituted in 50 μL of digestion buffer (100 mMTris-HCl pH 8.5, 1% SDC (Sigma-Aldrich), 5 mM TCEP and 30 mM CAA) to reduce and alkylate the proteins. Protein digestion was then performed by adding LysC at a 1:100 (w/w) ratio for 1 h at 37 °C, followed by overnight incubation at 37 °C with Trypsin at a 1:25 ratio (w/w). Digestions were quenched 0.5% FA, and the SDC precipitate was pelleted by centrifugation at 20,000 × *g* for 5 min at 4 °C. Supernatants were loaded twice into Pierce C18 10 µL bed Stage tips (Thermo Fisher) for desalting, washed with 100 μL of 0.1% FA, and peptides were eluted twice on 80% ACN, 0.1% FA, vacuum dried and stored at −80 °C before phosphopeptide enrichment.

### Automated Fe(III)-IMAC phosphopeptide enrichment

Phosphorylated peptides were enriched on Fe(III)-NTA 5 μL (Agilent technologies) cartridges using the AssayMAP Bravo Platform (Agilent Technologies). Fe(III)-NTA cartridges were primed with 250 μL of 0.1% TFA in ACN at a 100 μL/min flow rate and equilibrated with 250 μL of loading buffer (80% ACN/0.1% TFA) at a 50 μL/min flow rate. Samples were dissolved in 200 μL of loading buffer and loaded onto the cartridge at a 3 μL/min flow rate, followed by a 250 μL wash in loading buffer at 20 μL/min. The load flowthrough, containing the non-phosphorylated subset of the proteome, was kept for further proteomic characterization. The phosphopeptides were then eluted with 35 μL of 1% ammonia at 5 μL/min directly into 35 μL of 10% formic acid. Samples were vacuum dried and stored in −80 °C until LC–MS/MS analysis.

### LC–MS/MS analysis of EV-stimulated HMEC-1

Data were acquired with an Ultimate 3000 system (Thermo Fischer Scientific) coupled to an Orbitrap Exploris 480 mass spectrometer (Thermo Fischer Scientific). Peptides were trapped (Dr Maisch Reprosil C18, 3 µM, 2 cm × 100 µM) for 5 min in solvent A (0.1% formic acid in water) before being separated on an analytical column (Agilent Poroshell, EC-C18, 2.7 µM, 50 cm × 75 µM). Solvent B consisted of 0.1% formic acid in 80% acetonitrile. Trapping of peptides was performed for 2 min in 9% B at a flow rate of 300 nL/min. For full proteome analysis, peptides were separated in a gradient of 13–44% B in 95 min, while for phosphoproteomic analysis, peptides were separated in a gradient of 9–36% B in 36 min After (phospho)peptide separation, gradients were followed by a steep increase to 99% B in 3 min, a 5 min wash in 99% B and a 10 min re-equilibration at 9% B. Flow rate was kept at 300 nL/min. The mass spectrometer was operated in data-dependent mode. Peptides were ionized in a nESI source at 1.9 kV and focused at 40% amplitude of the RF lens. Full scan MS1 spectra from 375–1600 *m*/*z* were acquired in the Orbitrap at a resolution of 60,000 with the AGC target set to 1 × 10^6^ and under automated calculation of maximum injection time. Cycle time for MS2 fragmentation scans was set to 1 s. Only peptides with charged states 2–6 were fragmented, and dynamic exclusion was set to a duration of 10 ms for 36 min gradients and to 16 ms for 95 min gradients. Fragmentation was done using fixed HCD normalized collision energy of 28%. Fragment ions were accumulated until a target value of 1 × 10^5 ^ions was reached under an automated calculation of maximum injection time, with an isolation window of 1.4 *m*/*z* before injection in the Orbitrap for MS2 analysis at a resolution of 30,000. Proteomics raw data have been deposited to ProteomeXchange Consortium via the PRIDE repository^54^ and can be accessed through the identifier PXD030779.

### Database search and (phospho)proteomics data analysis

All (phospho)proteomics raw data were searched in MaxQuant (v_1.6.10.43)^55^ against the SwissProt human reference proteome database (containing 20,381 proteins and downloaded from Uniprot on March 2021). Spectra were searched using MaxQuant’s built-in Andromeda search engine. Trypsin was set as the digestion enzyme and up to two missed cleavages were allowed. Carbamidomethylation of cysteines was set as a fixed modification, while protein N-terminal acetylation and methionine oxidation were set as variable modifications. For phosphoproteomic data analysis, phosphorylation of serine, tyrosine and threonine were also included as variable modifications. Label-free quantification (LFQ) was enabled using a minimum ratio count of two and both razor and unique peptides for quantification. Match between runs was enabled, the matching time window was always set to 0.7 min while the alignment time window was set to 20 min for proteomic analysis and 10 min for phosphoproteomic analysis. Precursor ion tolerance was set to 20 ppm for the first search and 4.5 ppm after recalibration, and fragment ions tolerance was set to 20 ppm. False discovery rate (FDR) of 1% was set at PSM, site and protein level by using a reverse decoy database strategy. Data was analysed using Perseus software (v_1.6.14)^56^. In each analysis, proteins quantified (LFQ) in two out of three replicates were log2 transformed and missing values were replaced individually for each sample from the normal distribution. For phosphoproteomic analysis, where intensity-based MS injection balancing was not possible, phosphosite intensities were normalized to the maximum cumulative intensity (found in sample veh-EV, replicate 2). Statistical differences were always assessed by two-sided Student’s T-test or one-way ANOVA and corrected *p*-values (*q*-value) were calculated using the permutation method with up to 250 iterations. Proteins were considered significant when *q*-value ≤ 0.05. Gene ontology analysis were done using PANTHER^57^ with the human proteome as background gene set. All plots were generated using R packages^58^.

### Transmission electron microscopy (TEM)

Concentrated EVs were adsorbed to carbon-coated formvar grids for 15 min at RT. After a PBS wash, the grids were fixed in a 1% glutaraldehyde in PBS fixing buffer for 30 min at RT, followed by counterstaining with uranyl-oxalate. Grids were embedded in a mixture of 1.8% methyl cellulose and 0.4% uranyl acetate at 4 °C and imaged on a Jeol JEM-1011 TEM microscope (Jeol).

### Statistics and reproducibility

Statistical analyses of scratch assay, sprouting assay and western blot densitometry results were performed using Prism 5.0 (GraphPad Software Inc.). For most experiments, three biologically independent replicates were performed, and otherwise stated differently in the corresponding figure legend. The statistical difference between two groups was analysed using an unpaired Student’s T-test, and for densitometry analysis of WB results, with additional Welch’s correction. Differences between more than two groups were tested with a one-way ANOVA followed by Tukey’s HSD multiple comparison test as post-test. Differences with two-tailed *p*-values < 0.05 were considered statistically significant. All results are expressed as mean ± standard deviation (SD).

### Reporting summary

Further information on research design is available in the Nature Portfolio Reporting Summary linked to this article.

## Supplementary information

Supplementary Information

Description of Additional Supplementary Files

Supplementary Data 1

Reporting Summary

## Data Availability

The (phospho)proteomic datasets generated and analysed during the current study are available in the ProteomeXchange Consortium via the PRIDE repository^54^, and can be accessed through the identifier PXD030779. Numerical source data underlying all graphs and charts can be found in Supplementary Data 1.
