# Supplementary material for: Cardiac progenitor cell-derived extracellular vesicles promote angiogenesis through both associated- and co-isolated proteins
Source: Commun Biol. 2023 Aug 1;6:800. doi: 10.1038/s42003-023-05165-7 (PMC10393955; doi:10.1038/s42003-023-05165-7)
Supplement: Supplementary file 1 — Supplementary Information [file 42003_2023_5165_MOESM1_ESM.pdf]

Supplementary Information

Title: **Cardiac progenitor cell-derived extracellular vesicles promote angiogenesis through both associated- and co-isolated proteins**

Marieke Theodora Roefs[1], Julia Bauzá-Martinez[2,*], Simonides Immanuel van de Wakker[1,*], Jiabin Qin[1], Willem Theodoor Olijve[1], Robin Tuinte[1], Marjolein Rozeboom[1], Christian Snijders Blok[1], Emma Alise Mol[1], Wei Wu[2,3,*], Pieter Vader[1,4,*], Joost Petrus Gerardus Sluijter[1,*]

[1] Department of Experimental Cardiology, University Medical Center Utrecht, Utrecht University, The Netherlands.

[2] Biomolecular Mass Spectrometry and Proteomics, Bijvoet Center for Biomolecular Research and Utrecht Institute for Pharmaceutical Sciences, Utrecht University, Utrecht, The Netherlands.

[3] Singapore Immunology Network (SIgN), ASTAR (Agency for Science, Technology and Research), Singapore, Singapore.

[4] CDL Research, University Medical Center Utrecht, The Netherlands.

Supplementary Table 1

Sequences of all guide RNAs for each CRISPR

		Genomic target region
PAPP-A gRNA 1	5'-AGGAGTAGCAACTTGGCCAT-3'	exon 3
PAPP-A gRNA 2	5'-AGTTGGCAGGAGTAGCAACT-3'	exon 3
PAPPA-A gRNA 3	5'-GGCCTCTATCACGTCTTCCG-3'	exon 4
NID1 gRNA 1	5'-GGGTTTGCGGGACCGCAGTT-3'	5'UTR/exon1
NID1 gRNA 2	5'-GAACTGCGGTCCCGCAAACC-3'	5'UTR
NID1 gRNA 3	5'-TGTCGGATCTGTCGTAGAAG-3'	exon1
Non targeting	5'-ATTTCCCTACGGAGATATCC-3'	

Primer sequences of genomic DNA amplification for T7 endonuclease assay

PAPP-A exon 3	F: 5'-GCCTGTTATCTTTTTGGGGGC-3'
	R: 5'-CTGTGCCAGTACAGTGTGGT-3'
PAPPA-A exon 4	F: 5'-GCTCTCAGGGCAATGGAGTC-3'
	R: 5'-AACCCAGAAAGAGAATGACCGA-3'
NID1 exon 1	F: 5'-AACAAGTTGACAGCGACCCC-3'
	R: 5'-CCGGTTACATCCCCGCCTTC-3'

Supplementary Table 2

Top-20 proteins (of n=105) enriched in veh-EVs compared with Ca ion-EVs and SKOV-3-EVs. Ranked by the mean protein abundance (log2) in veh-EVs, the top 20 significantly enriched (q-value of Student's T-test) proteins are displayed.

**Gene name**	**Mean veh-EVs**	**Mean CaIon-EVs**	**Mean SKOV-3-EVs**	**q-value (Student's T-test) veh-EVs *vs.* CaIon-EVs**	**q-value (Student's T-test) veh-EVs *vs.* SKOV-3-EVs**
**COL1A1**	31.2	23.7	19.2	0.0228	0.0056
**COL6A3**	30.9	24.9	19.4	0.0013	0.0045
**FN1**	29.9	26.6	26.5	0.0171	0.0059
**LAMA4**	29.5	23.3	19.7	0.0053	0.0000
**LAMC1**	29.4	24.8	27.1	0.0000	0.0032
**COL1A2**	29.3	22.0	19.8	0.0241	0.0060
**APP**	29.3	22.1	21.8	0.0009	0.0054
**UBA52**	29.1	27.4	27.7	0.0122	0.0249
**VCAN**	29.0	25.2	18.9	0.0050	0.0055
**EDIL3**	29.0	24.3	17.6	0.0029	0.0000
**LAMB1**	28.8	24.2	26.5	0.0000	0.0030
**COL6A1**	28.8	23.9	22.8	0.0049	0.0043
**SRGN**	28.2	18.3	19.3	0.0051	0.0066
**COL6A2**	28.1	22.1	18.6	0.0047	0.0000
**PAPPA**	28.1	21.5	16.7	0.0051	0.0000
**ANPEP**	28.0	24.6	17.8	0.0118	0.0000
**THBS1**	27.9	23.9	26.4	0.0030	0.0200
**NID1**	27.8	19.2	19.7	0.0000	0.0049
**BASP1**	27.7	25.5	23.7	0.0050	0.0057
**ITGB1**	27.6	25.4	26.3	0.0175	0.0068

Supplementary Figures

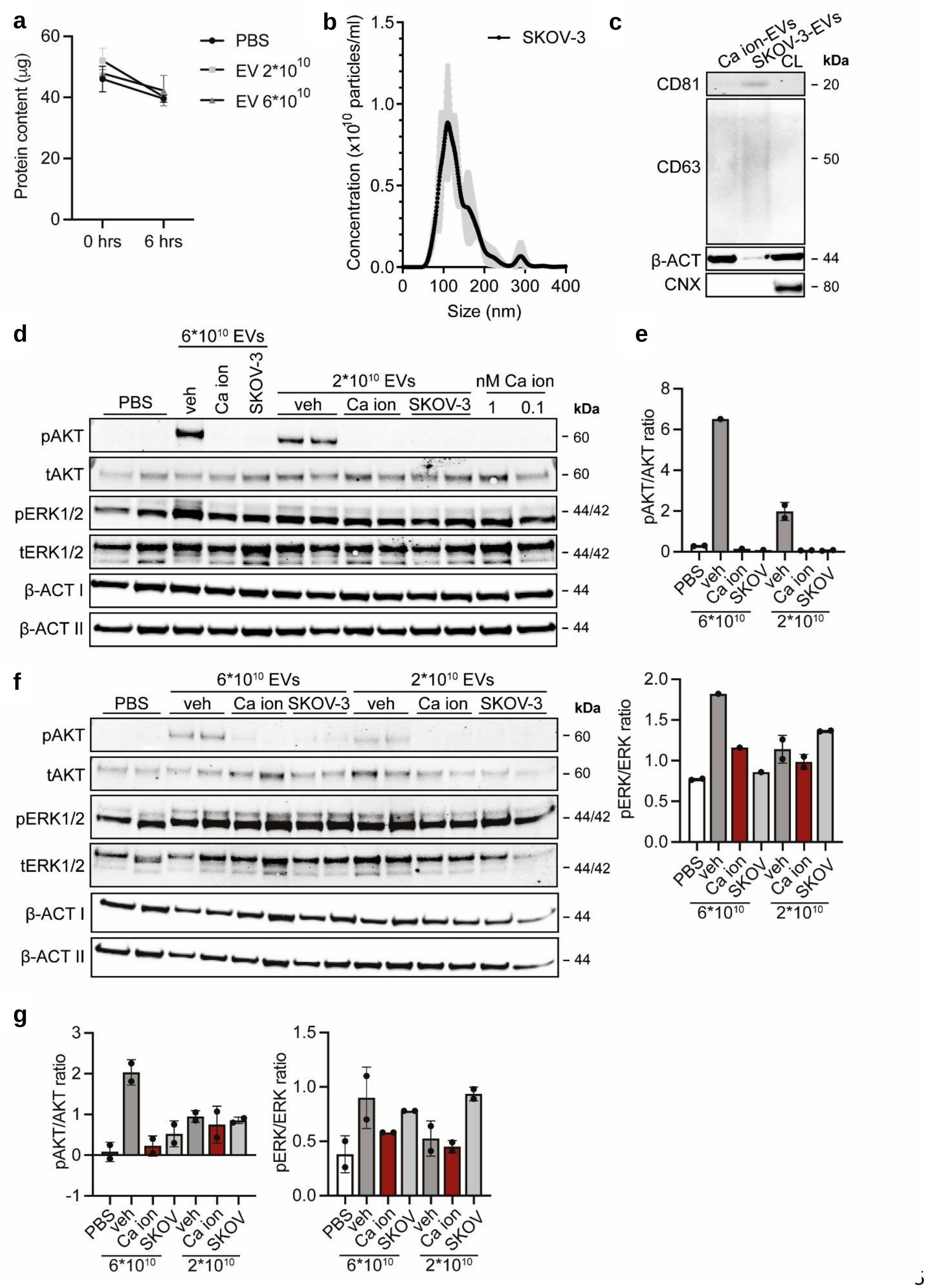
a
Protein content (µg)
0 hrs
6 hrs
PBS
EV 2*10^10
EV 6*10^10
b
Concentration (x10^10 particles/ml)
Size (nm)
SKOV-3
c
Ca ion-EVs
SKOV-3-EVs
CL
kDa
CD81
CD63
β-ACT
CNX
d
6*10^10 EVs
PBS
veh
Ca ion
SKOV-3
2*10^10 EVs
nM Ca ion
pAKT
tAKT
pERK1/2
tERK1/2
β-ACT I
β-ACT II
e
pAKT/AKT ratio
f
pERK/ERK ratio
g
pAKT/AKT ratio
pERK/ERK ratio

Supplementary Figure 1. SKOV-3 EVs do not activate intracellular signaling in HMEC-1. (a) Total protein content of HMEC-1 lysates collected 0 or 6 hrs after stimulation with PBS, $2*10^{10}$ or $6*10^{10}$ EVs. Data are displayed as mean ±SD (n=3). (b) Representative NTA plot showing the size distribution and particle concentration of SKOV-3-EVs. (c) Western blot analysis showing the presence of CD81, CD63, β-actin (β-ACT), and absence of Calnexin (CNX) in Ca ion-EVs and SKOV-3-EVs. β-ACT and CNX were present in Ca ion-CPC lysate (CL). (d, e) Representative western blot analysis of phosphorylated AKT (pAKT), total AKT (tAKT), phosphorylated ERK1/2 (pERK1/2) and total ERK1/2 (tERK1/2) in HMEC-1 treated with veh-, Ca ion- and SKOV-3-EVs normalized on two doses of EV particle numbers, and with 1 nM and 0.1 nM calcium ionophore A23187 in PBS. β-ACT was included as housekeeping protein. (e) Quantification of pAKT, tAKT, pERK1/2 and tERK1/2 expression levels using densitometry expressed as pAKT/AKT and pERK/ERK ratios. (f, g) Biological replicate of (d) and (e). Data are presented as mean ± SD.

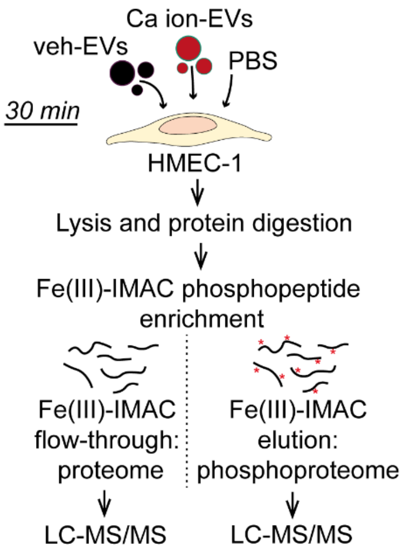

Supplementary Figure 2. Workflow used for phosphoproteomic analysis. Schematic of workflow used for phosphoproteomic analysis by liquid chromatography-mass spectrometry (LC-MS/MS). EV stimulated HMEC-1 were lysed and extracted proteins were digested into peptides and loaded into Fe(III)-Immobilized Metal Affinity Chromatography (Fe(III)-IMAC) cartridges for phosphopeptide enrichment. Fe(III)- IMAC flow-through containing the non-phosphorylated subset of proteome, and elutions containing the phosphorylated subset of the proteome were analysed by (LC-MS/MS).

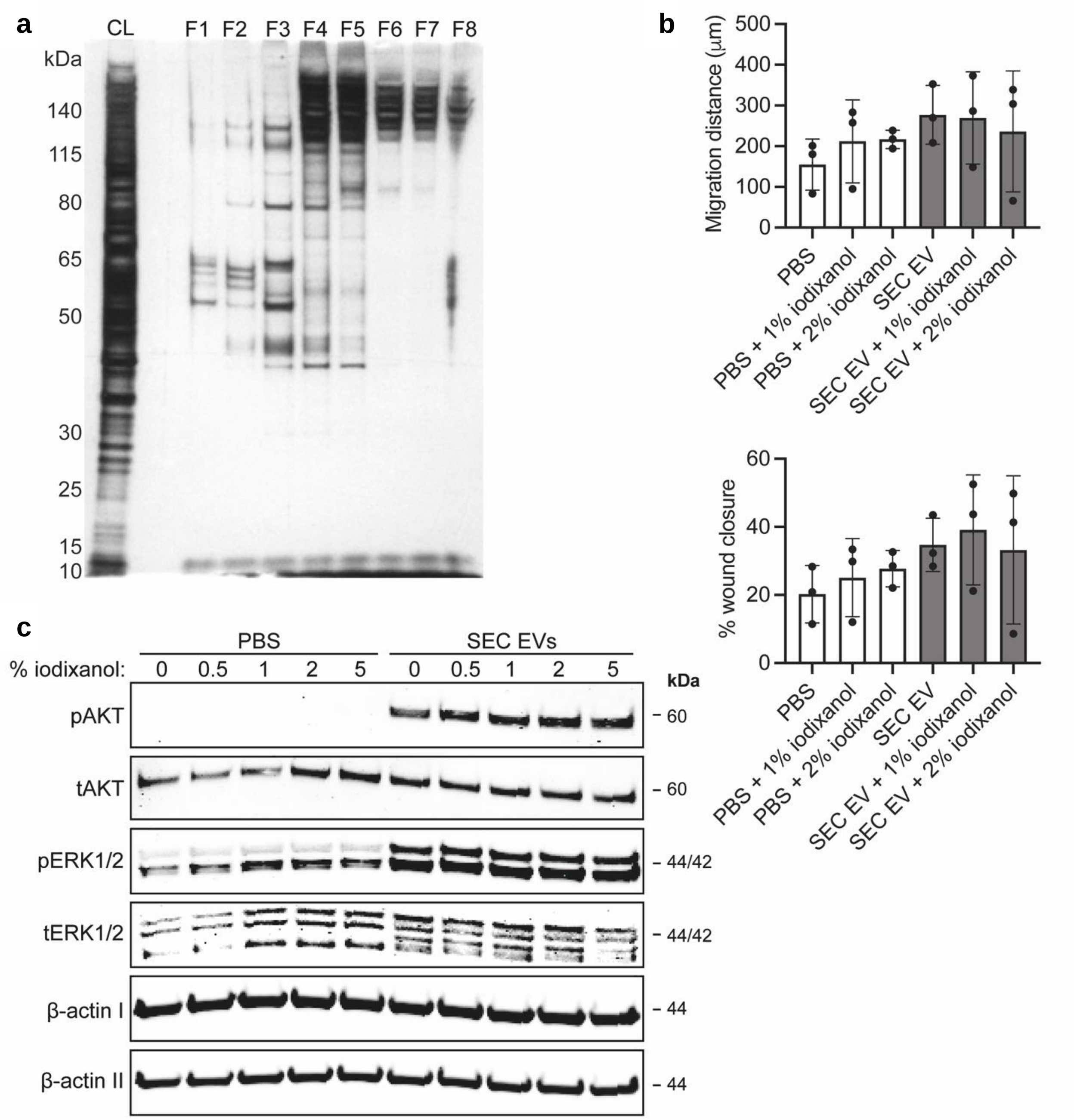

Supplementary Figure 3. Influence of iodixanol concentration on EV-mediated HMEC-1 activation. (a) Silver stain of fractions (F1-8) collected after ultracentrifugation of SEC-EVs loaded in the bottom of a discontinuous Optiprep^{TM} gradient. Equal volumes of each sample were analysed, and 1 µg CPC lysate (CL) was included as control. (b) Representative wound healing assay showing effects of PBS and 1 µg CPC-EVs simultaneous with different concentrations of iodixanol on HMEC-1 migration, analysed both as absolute migration distance (top) and % wound closure (bottom) (n=3, technical replicates. Data are

representative of three biologically independent experiments). Data are presented as mean ± SD. (c) Representative western blot analysis of phosphorylated AKT (pAKT), total AKT (tAKT), phosphorylated ERK1/2 (pERK1/2) and total ERK1/2 (tERK1/2) in HMEC-1 treated with PBS and $2x10^{10}$ EVs. simultaneous with different concentrations of iodixanol (0-5%). β-ACT was included as housekeeping protein.

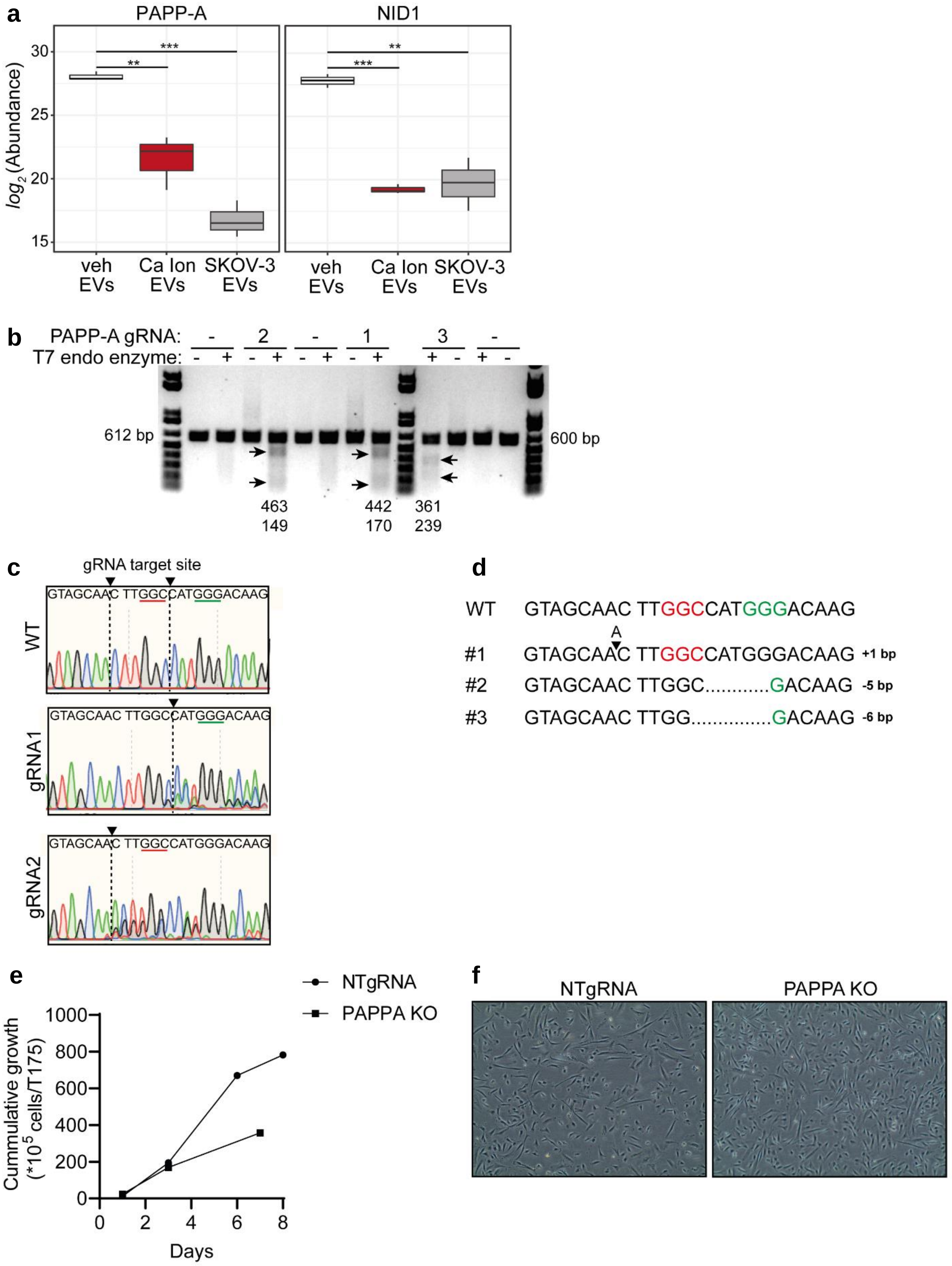

a
PAPP-A
NID1
log2 (Abundance)
30
25
20
15
***
**
veh EVs
Ca Ion EVs
SKOV-3 EVs
b
PAPP-A gRNA: - 2 - 1 3 -
T7 endo enzyme: - + - + - + - + + - + -
612 bp
600 bp
463
149
442
170
361
239
c
gRNA target site
GTAGCAAC TTGGCCATGGGACAAG
WT
gRNA1
gRNA2
d
WT GTAGCAAC TTGGCCATGGGACAAG
A
#1 GTAGCAAC TTGGCCATGGGACAAG +1 bp
#2 GTAGCAAC TTGGC............GACAAG -5 bp
#3 GTAGCAAC TTGG...............GACAAG -6 bp
e
NTgRNA
PAPPA KO
Cummulative growth (*10^5 cells/T175)
1000
800
600
400
200
0
0 2 4 6 8
Days
f
NTgRNA
PAPPA KO

Supplementary Figure 4. Generation of PAPPA knock-out CPC lines. (a) Boxplots representing protein abundance (log_2) of PAPP-A and NID1 in veh-, Ca ion- and SKOV-3-EVs, as measured with by LC-MS/MS. Corrected P-values were calculated using student's T-test. ** = q<0.01. *** = q<0.001. Boxes represent the 25%, 50% (median) and 75% quartiles and whiskers represent the ±1.5 interquartile range. (b) T7 endonuclease assay confirming a double-stranded break at the CRISPR/Cas9 target site in exon 3 of *PAPPA* in CPCs transduced with three different gRNAs (1-3). (c) Sanger sequencing results confirming frameshift in *PAPPA* exon 3 at the CRISPR/Cas9 target site of gRNA1 and gRNA2, compared with wild-type (WT) CPC genomic DNA. (d) Genomic DNA sequence surrounding two gRNA target sites in exon 3 of *PAPPA* of three individual PAPPA KO clones. Clones had homozygous mutations on both alleles. (e) Growth curves of NTgRNA- and PAPPA KO-CPC lines selected for functional studies, as visualized as cumulative cell growth. (f) Cell viability of PAPPA KO- and NTgRNA-CPC clones.

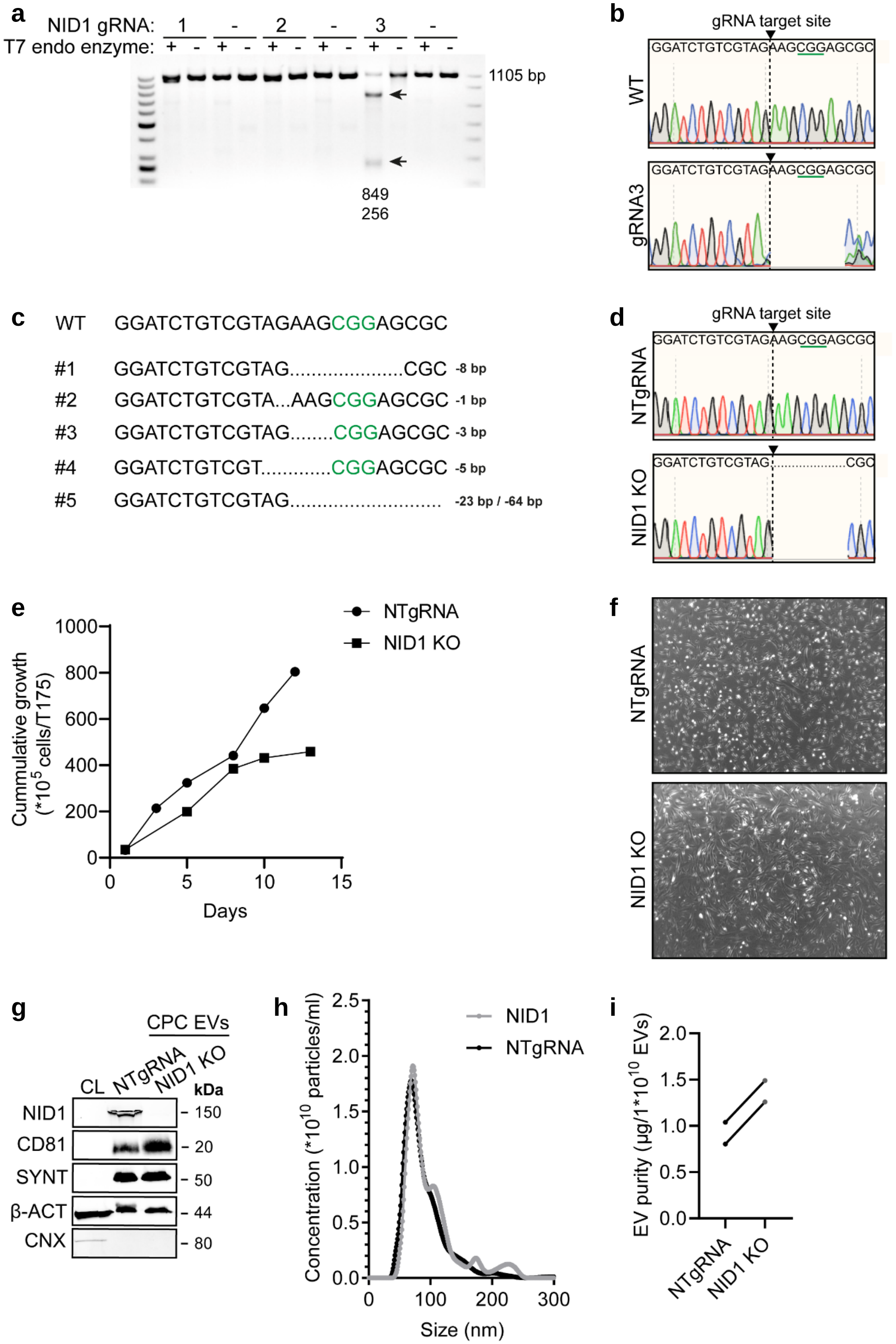
a
NID1 gRNA: 1 - 2 - 3 -
T7 endo enzyme: + - + - + - + - + - + -
1105 bp
849
256
b
gRNA target site
WT
gRNA3
GGATCTGTCGTAGAAGCGGAGCGC
c
WT GGATCTGTCGTAGAAGCGGAGCGC
#1 GGATCTGTCGTAG....................CGC -8 bp
#2 GGATCTGTCGTA...AAGCGGAGCGC -1 bp
#3 GGATCTGTCGTAG........CGGAGCGC -3 bp
#4 GGATCTGTCGT............CGGAGCGC -5 bp
#5 GGATCTGTCGTAG........................... -23 bp / -64 bp
d
gRNA target site
NTgRNA
NID1 KO
GGATCTGTCGTAG...................CGC
e
NTgRNA
NID1 KO
Cummulative growth (*10^5 cells/T175)
Days
f
NTgRNA
NID1 KO
g
CPC EVs
CL NTgRNA NID1 KO
kDa
NID1 150
CD81 20
SYNT 50
β-ACT 44
CNX 80
h
NID1
NTgRNA
Concentration (*10^10 particles/ml)
Size (nm)
i
EV purity (μg/1*10^10 EVs)
NTgRNA NID1 KO

Supplementary Figure 5. Generation of NID1 knock-out CPC lines. (a) T7 endonuclease assay confirming a double-stranded break at the CRISPR/Cas9 target site in exon 1 of *NID1* in CPCs transduced with three different gRNAs (1-3). (b) Sanger sequencing results confirming frameshift in *NID1* exon 1 at the CRISPR/Cas9 target site of gRNA3, compared with wild-type (WT) CPC genomic DNA. (c) Genomic DNA sequence surrounding the gRNA3 target site in exon 1 of *NID1* of five individual NID1 KO clones. Clones had homozygous mutations on both alleles. (d) Sanger sequencing results confirming 8 bp deletion in exon 1 of *NID1* at the CRISPR/Cas9 target site of the NID1 KO-CPC clone, compared with the NTgRNA polyclonal CPC line. (e) Growth curves of NTgRNA- and NID1 KO-CPC lines selected for functional studies, as visualized as cumulative cell growth. (f) Cell viability of NID1 KO- and NTgRNA-CPC clones. (g) Western blot analysis showing the absence of NID1 in NID1 KO-EVs compared with NTgRNA-EVs; the presence of CD81, Syntenin-1 (SYNT), β-actin (β-ACT), and absence of Calnexin (CNX) in both EV populations. β-ACT and CNX were present in CPC lysate (CL). (h) Representative NTA plot showing the size distribution and particle concentration of NID1 KO- and NTgRNA-CPC-EVs. (i) Protein content per $1x10^{10}$ NID1 KO- and NTgRNA-EVs of two representative experiments.

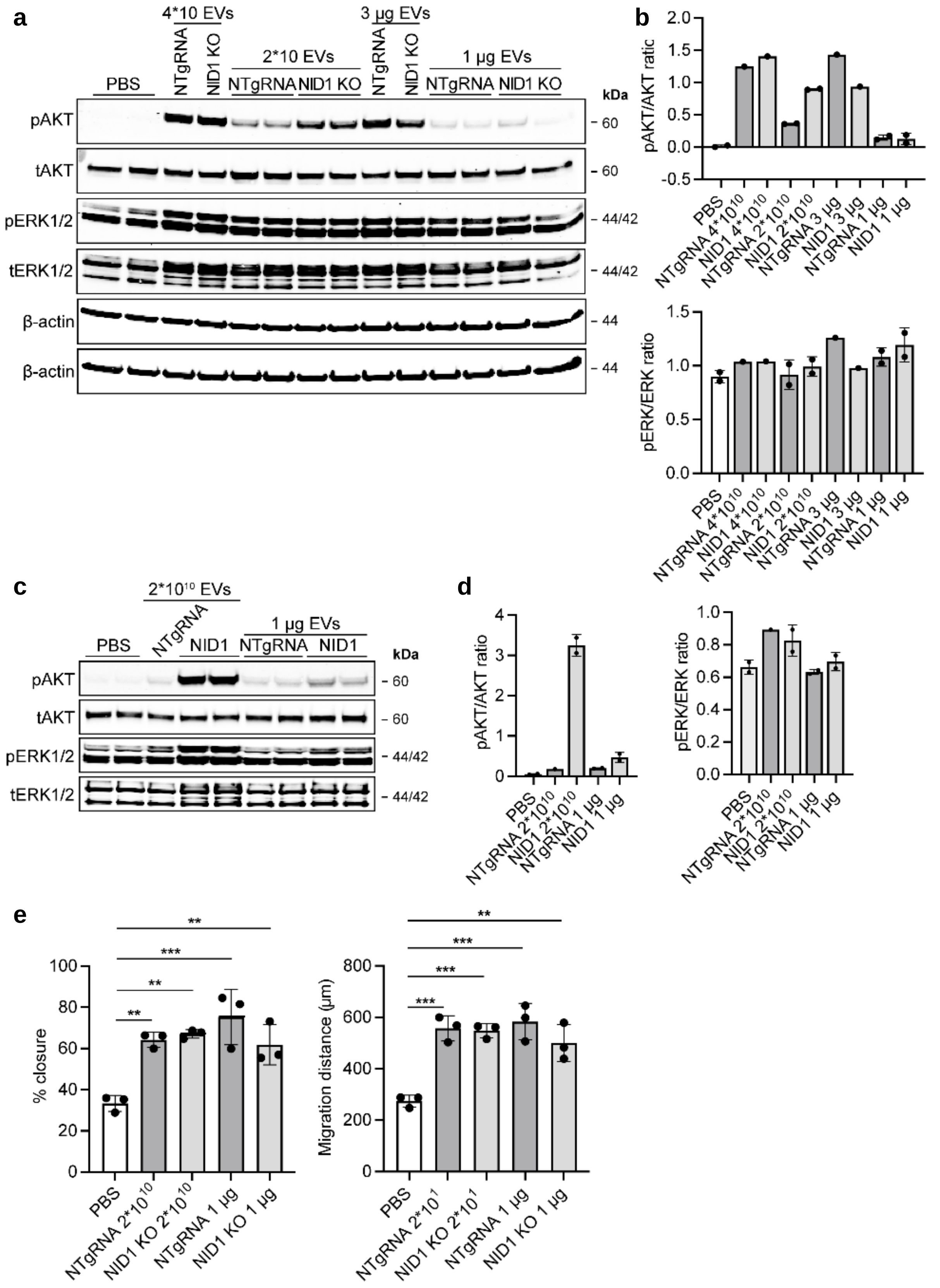

a
4*10 EVs
3 µg EVs
2*10 EVs
1 µg EVs
PBS
NTgRNA
NID1 KO
kDa
pAKT
tAKT
pERK1/2
tERK1/2
β-actin
- 60
- 44/42
- 44
b
pAKT/AKT ratio
pERK/ERK ratio
PBS
NTgRNA 4*10^10
NID1 4*10^10
NTgRNA 2*10^10
NID1 2*10^10
NTgRNA 3 µg
NID1 3 µg
NTgRNA 1 µg
NID1 1 µg
c
2*10^10 EVs
1 µg EVs
NID1
d
e
% closure
Migration distance (µm)
NID1 KO 2*10^10
NTgRNA 2*10^10
NID1 KO 1 µg
**
***

Supplementary Figure 6. NID1 KO-EVs were as potent as NTgRNA-EVs in activating intracellular signalling in HMEC-1 and in inducing HMEC-1 migration. (a) Representative western blot analysis of phosphorylated AKT (pAKT), total AKT (tAKT), phosphorylated ERK1/2 (pERK1/2) and total ERK1/2 (tERK1/2) in HMEC-1 treated with NID1 KO- and NTgRNA-EVs normalized on two doses of EV particle numbers or EV total protein content. β-actin (β-ACT) was included as housekeeping protein. (b) Quantification of pAKT, tAKT, pERK1/2 and tERK1/2 expression levels using densitometry expressed as pAKT/AKT and pERK/ERK ratios. (c, d) Biological replicate of (a) and (b). (e) Representative wound healing experiment showing effects of $2x10^{10}$ and 1 µg NTgRNA- and NID1 KO-EVs on HMEC-1 migration, analysed both as % wound closure and absolute migration distance (n=3, technical replicates. Data are representative of biologically independent experiments). Data are presented as mean ± SD. **= $p < 0.0021$. ***= $p < 0.0002$.

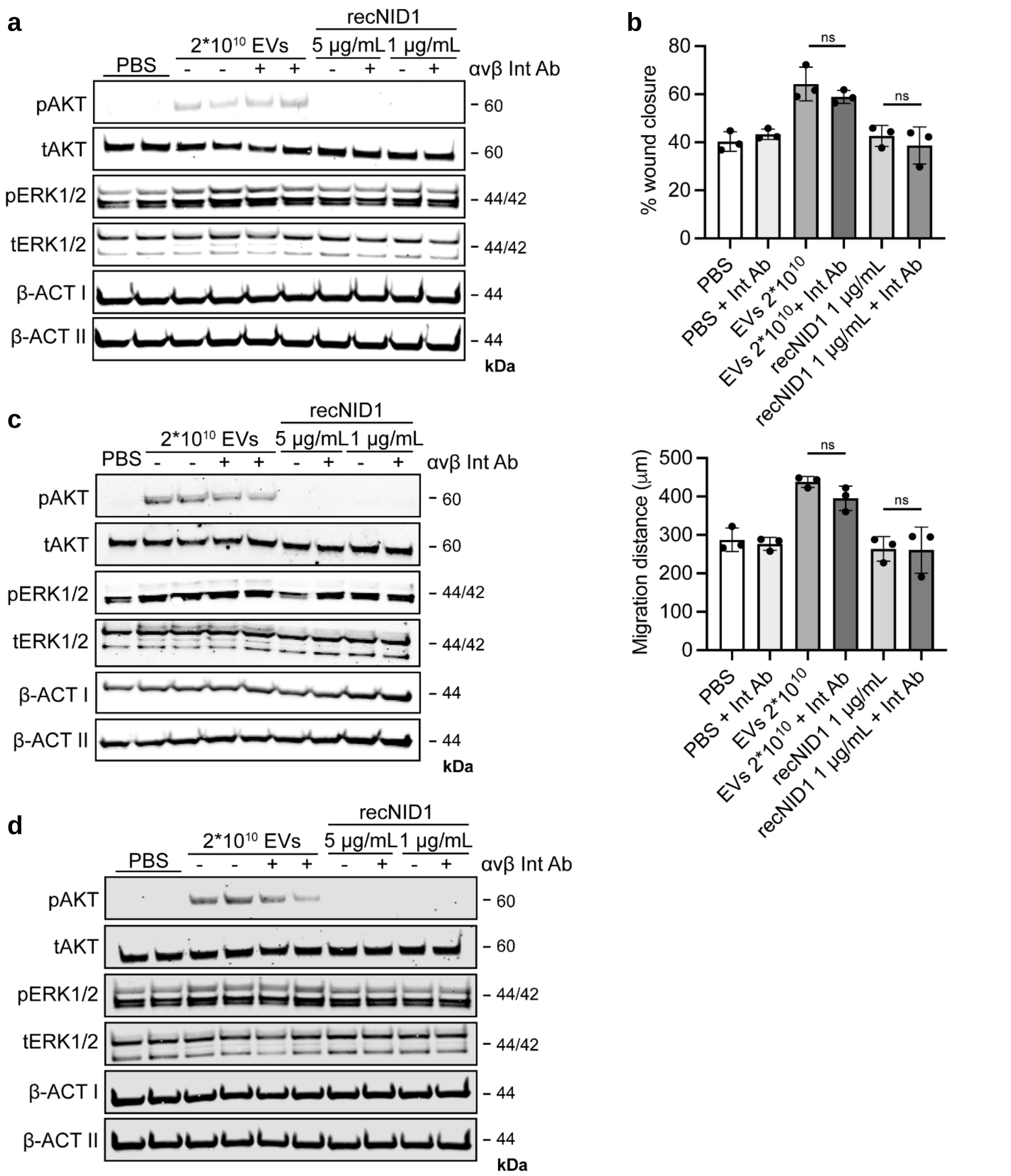

Supplementary Figure 7. Influence of IntegrinαVβ3 blocking on HMEC-1 activation and migration. (a) Representative western blot analysis of phosphorylated AKT (pAKT), total AKT (tAKT), phosphorylated ERK1/2 (pERK1/2) and total ERK1/2 (tERK1/2) in HMEC-1 treated with 2x10^{10} CPC-EVs, or with 5 µg/mL

or 1 µg/mL recombinant IGF-1 after pre-incubation with (+) or without (-) 10 µg/mL IntegrinαVβ3 antibody. β-actin (β-ACT) was included as housekeeping protein. (b) Representative wound healing assay showing effects of 10 µg/mL IntegrinαVβ3 antibody addition to CPC-EV-induced HMEC-1 migration, analysed both as % wound closure and absolute migration distance. 5 µg/mL and 1 µg/mL recombinant NID1 (recNID1) were included as control (n=3, technical replicates. Data are representative of biologically independent experiments). (c, d) Biological replicates of (a). Data are presented as mean ± SD. ns= not-significant.

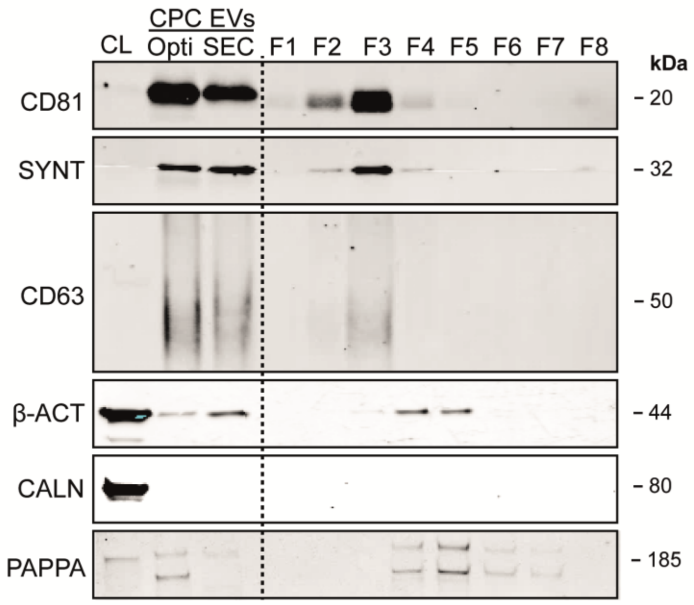

Supplementary Figure 8. PAPP-A presence in iodixanol gradient fractions.

SEC-EVs were separated in a discontinuous Optiprep™ gradient and resulting fractions (F1-8) were analysed for the presence of EV-marker proteins CD81, CD63, Syntenin-1 (SYNT), β-actin (β-ACT) and PAPP-A and absence of Calenexin (CALN) by western blotting. Equal volumes of each sample were analysed. Cell lysate (CL) and Opti-EVs, retrieved by pooling and concentration of fractions 1-5 using a 100 kDa cut-off spin filter, were included. CD81, CD63 and SYNT blots are also displayed in *Figure 8b.*

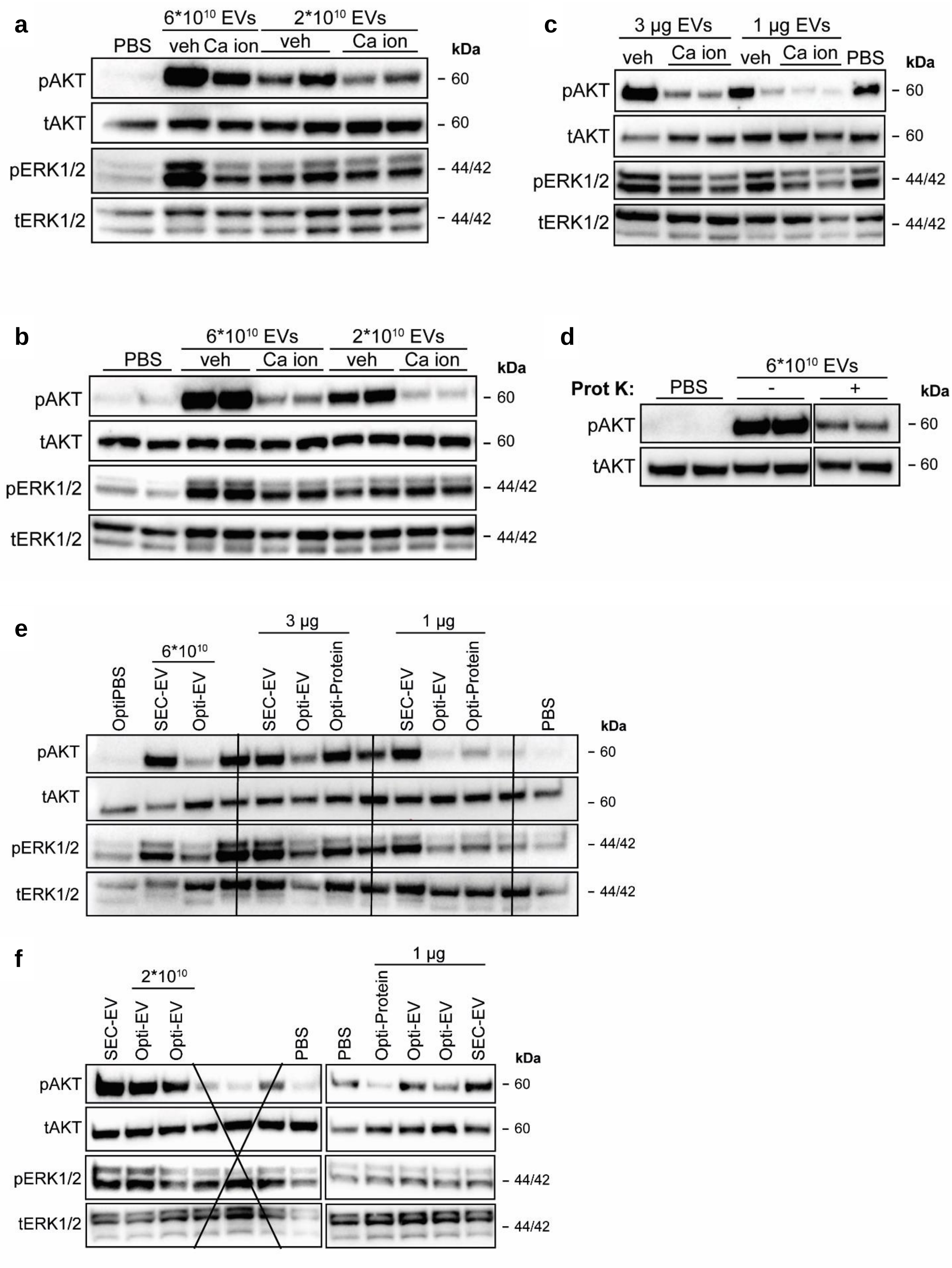
a
PBS
6*10^{10} EVs
veh
Ca ion
2*10^{10} EVs
veh
Ca ion
kDa
pAKT
tAKT
pERK1/2
tERK1/2
- 60
- 60
- 44/42
- 44/42
b
PBS
6*10^{10} EVs
veh
Ca ion
2*10^{10} EVs
veh
Ca ion
kDa
pAKT
tAKT
pERK1/2
tERK1/2
- 60
- 60
- 44/42
- 44/42
c
3 µg EVs
veh
Ca ion
1 µg EVs
veh
Ca ion
PBS
kDa
pAKT
tAKT
pERK1/2
tERK1/2
- 60
- 60
- 44/42
- 44/42
d
Prot K:
PBS
6*10^{10} EVs
-
+
kDa
pAKT
tAKT
- 60
- 60
e
OptiPBS
6*10^{10}
SEC-EV
Opti-EV
3 µg
SEC-EV
Opti-EV
Opti-Protein
1 µg
SEC-EV
Opti-EV
Opti-Protein
PBS
kDa
pAKT
tAKT
pERK1/2
tERK1/2
- 60
- 60
- 44/42
- 44/42
f
SEC-EV
2*10^{10}
Opti-EV
Opti-EV
PBS
PBS
1 µg
Opti-Protein
Opti-EV
Opti-EV
SEC-EV
kDa
pAKT
tAKT
pERK1/2
tERK1/2
- 60
- 60
- 44/42
- 44/42

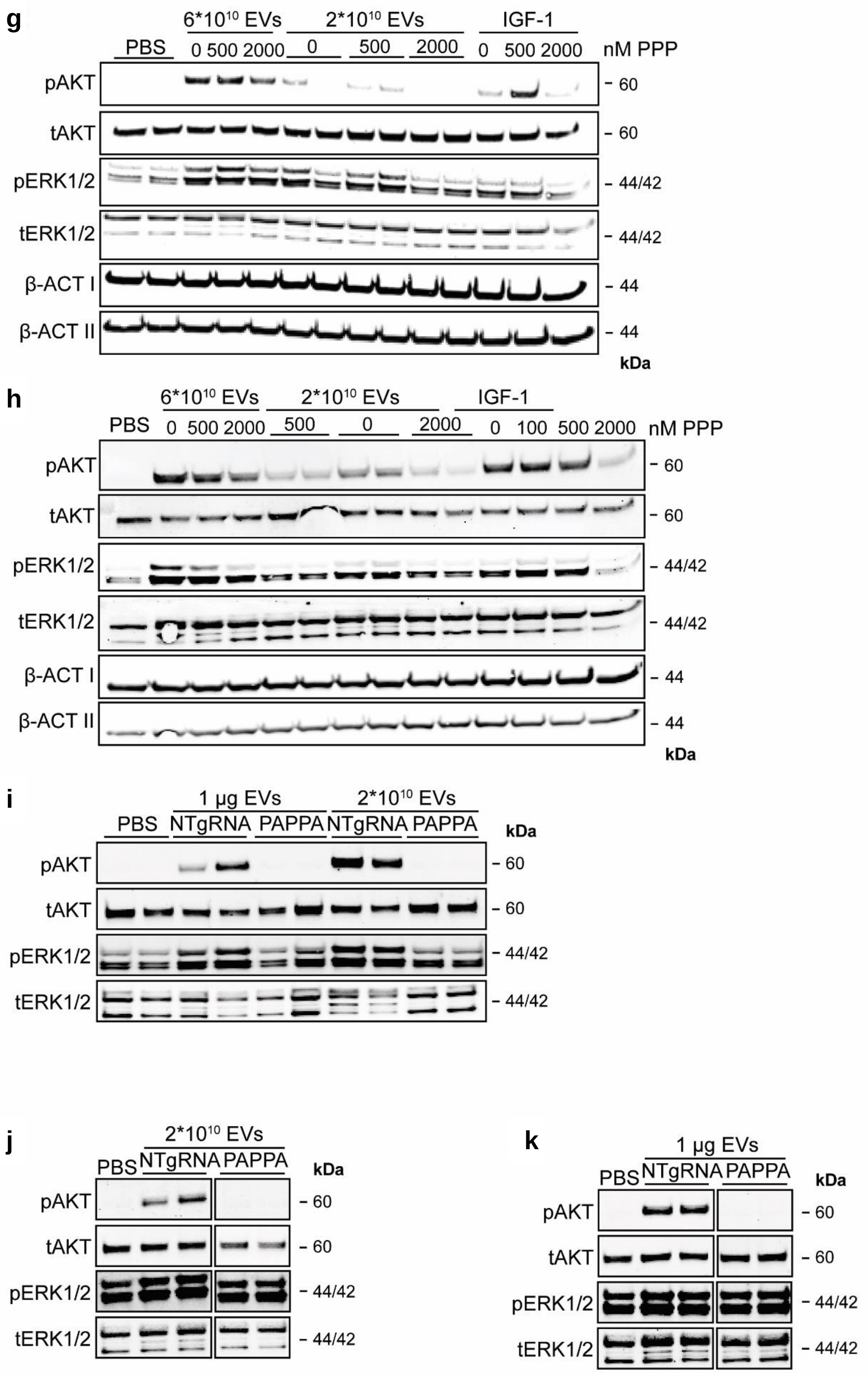
g
6*10^10 EVs
2*10^10 EVs
IGF-1
PBS
0 500 2000
0
500
2000
0 500 2000
nM PPP
pAKT
tAKT
pERK1/2
tERK1/2
β-ACT I
β-ACT II
- 60
- 60
- 44/42
- 44/42
- 44
- 44
kDa
h
6*10^10 EVs
2*10^10 EVs
IGF-1
PBS
0 500 2000
500
0
2000
0 100 500 2000
nM PPP
pAKT
tAKT
pERK1/2
tERK1/2
β-ACT I
β-ACT II
- 60
- 60
- 44/42
- 44/42
- 44
- 44
kDa
i
1 μg EVs
2*10^10 EVs
PBS
NTgRNA
PAPPA
NTgRNA
PAPPA
kDa
pAKT
tAKT
pERK1/2
tERK1/2
- 60
- 60
- 44/42
- 44/42
j
2*10^10 EVs
PBS
NTgRNA
PAPPA
kDa
pAKT
tAKT
pERK1/2
tERK1/2
- 60
- 60
- 44/42
- 44/42
k
1 μg EVs
PBS
NTgRNA
PAPPA
kDa
pAKT
tAKT
pERK1/2
tERK1/2
- 60
- 60
- 44/42
- 44/42

Supplementary Figure 9. Biological replicates of western blot analysis and quantification of pAKT, AKT, pERK and ERK expression levels. Biological replicates of Figure: (a, b) 2e; (c) 2g; (d) 4d; (e-f) 8g; (g, h) 6b; (i-k) 7a, c.

Figure 1b

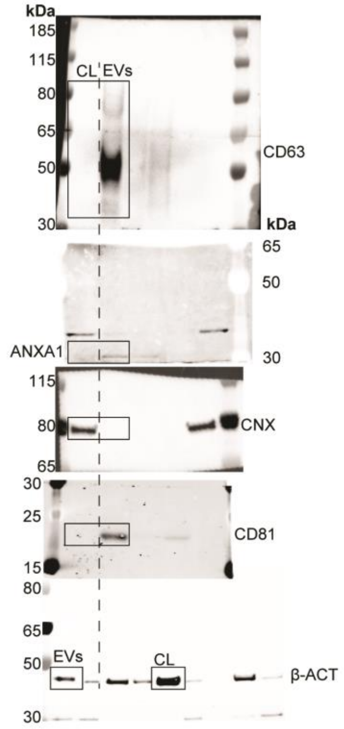

Figure 1e

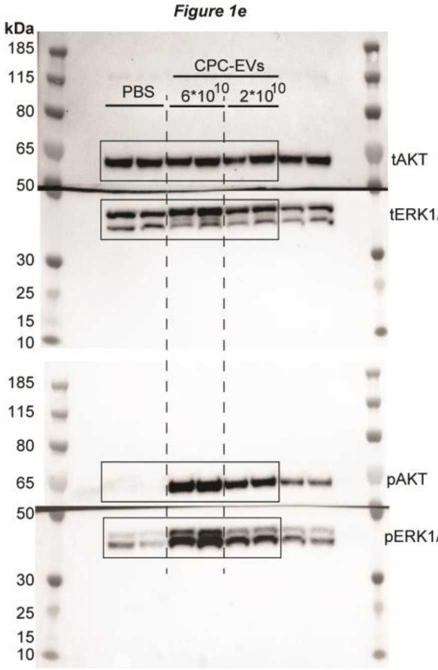

Figure 2c

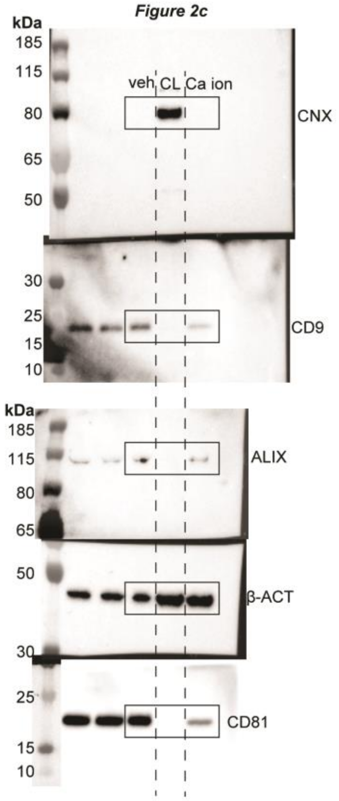

Figure 2e

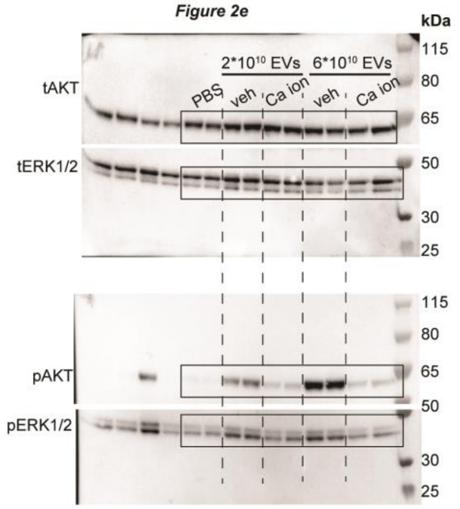

Figure 2g

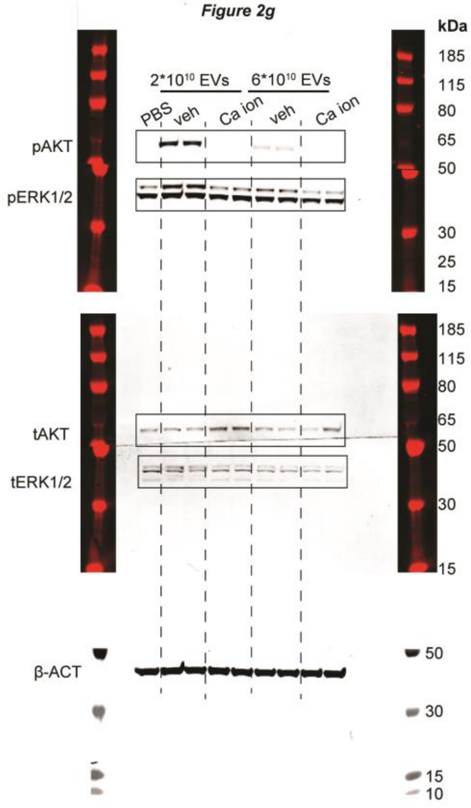

Figure 4c

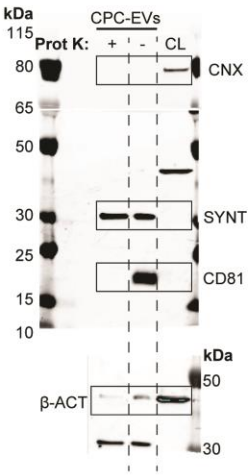

Figure 4d

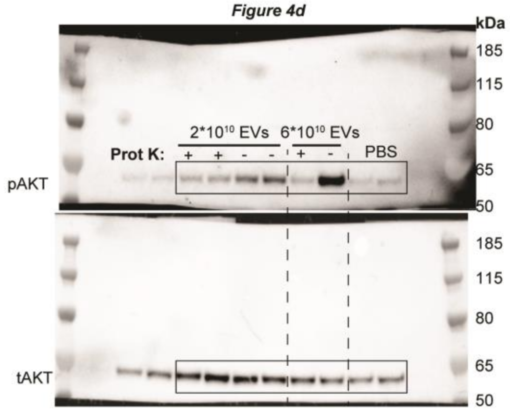

Figure 5c

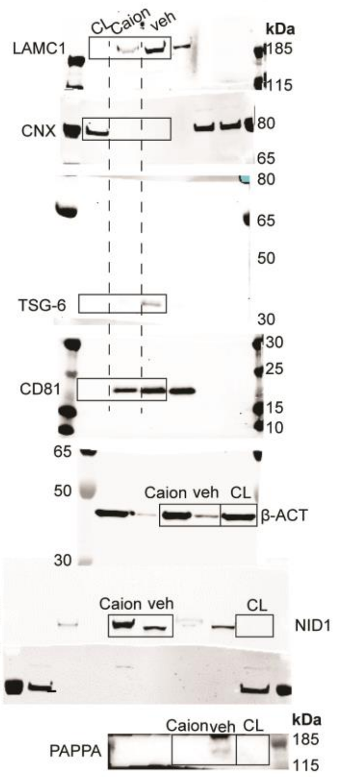

Figure 8b

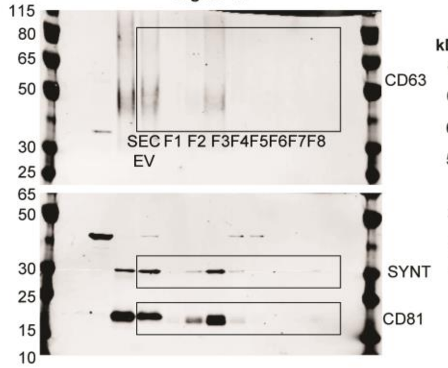

Figure 8e

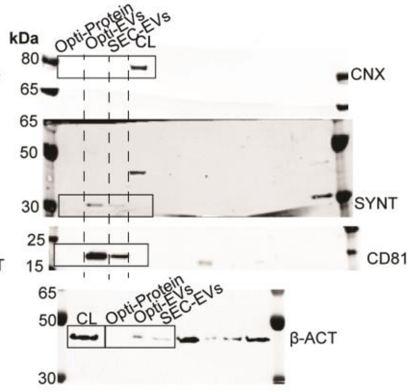

Figure 6d

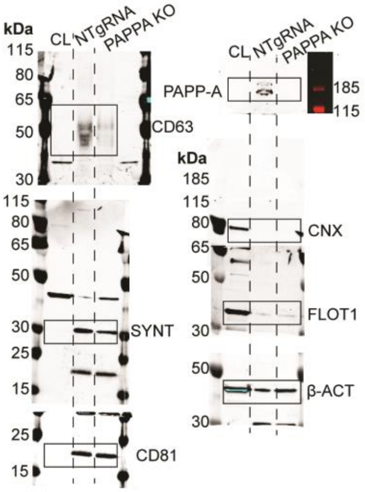

Figure 8g

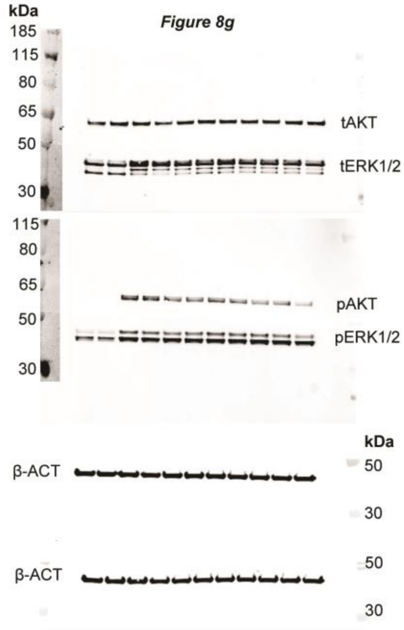

Figure 6b

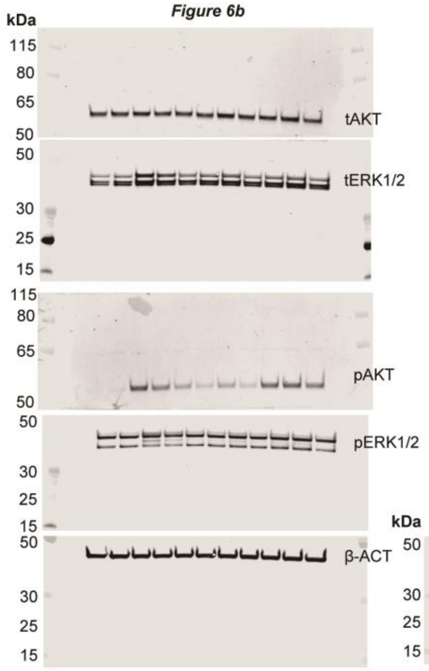

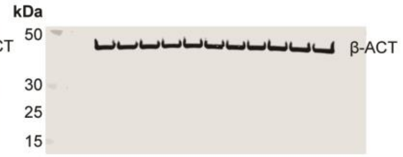

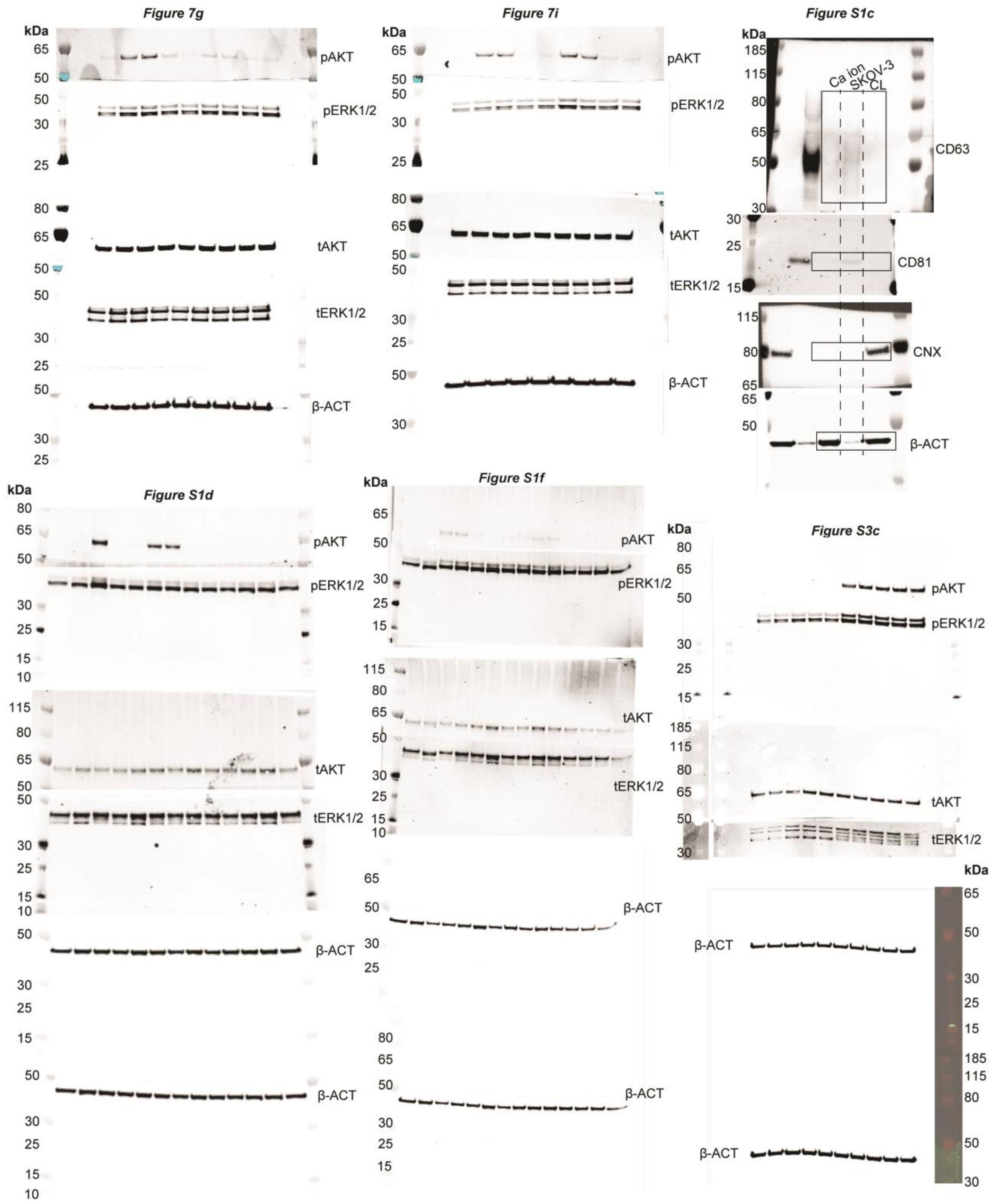
Figure 7g
kDa
65
50
pAKT
50
30
25
pERK1/2
80
65
50
tAKT
50
30
25
tERK1/2
50
β-ACT
30
25
Figure 7i
kDa
65
50
pAKT
50
30
25
pERK1/2
80
65
50
tAKT
50
tERK1/2
30
25
50
β-ACT
30
Figure S1c
kDa
185
115
80
65
50
30
Ca ion
SKOV-3
CL
CD63
30
25
15
CD81
115
80
65
CNX
65
50
β-ACT
Figure S1d
kDa
80
65
50
pAKT
pERK1/2
30
25
15
10
115
80
65
50
tAKT
50
tERK1/2
30
25
15
10
50
β-ACT
30
25
15
50
β-ACT
30
25
15
10
Figure S1f
kDa
65
50
pAKT
30
25
15
pERK1/2
115
80
65
50
tAKT
30
25
15
10
tERK1/2
65
50
β-ACT
30
25
80
65
50
β-ACT
30
25
15
Figure S3c
kDa
80
65
50
pAKT
pERK1/2
30
25
15
185
115
80
65
50
tAKT
30
tERK1/2
β-ACT
β-ACT
kDa
65
50
30
25
15
185
115
80
50
30

Figure S5g

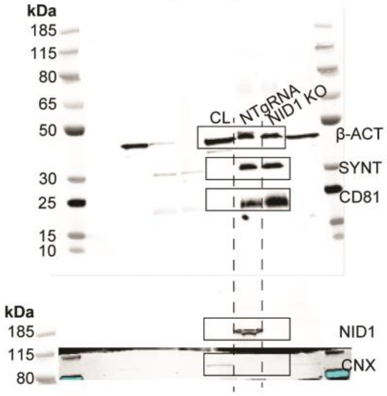

Figure S6a

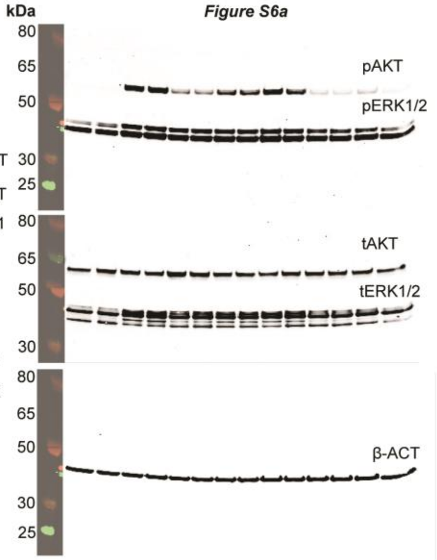

Figure S6c

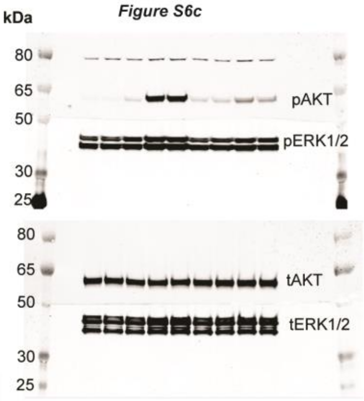

Figure S7a

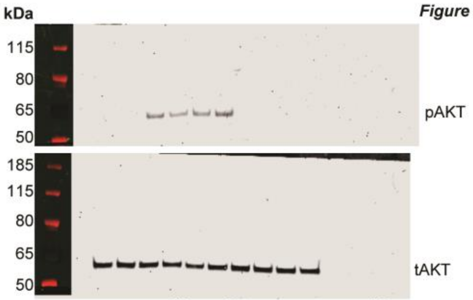

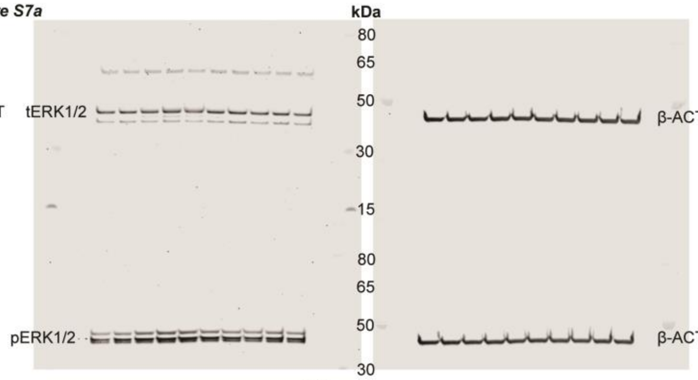

Figure S7c

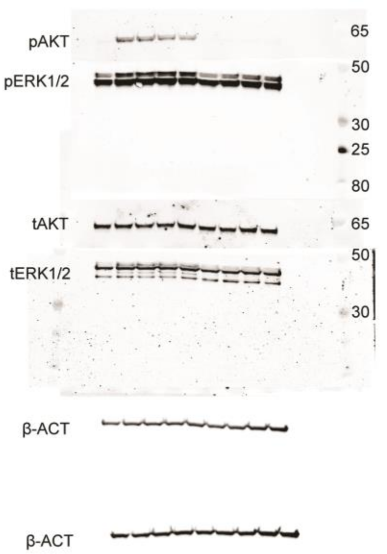

Figure S7d

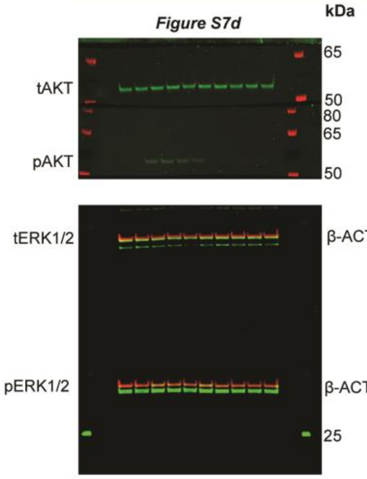

Figure S9a

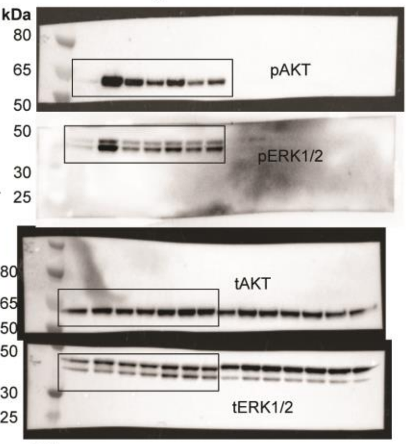

Figure S8

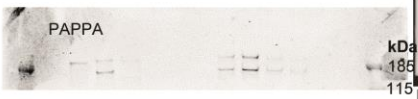

Figure S9b

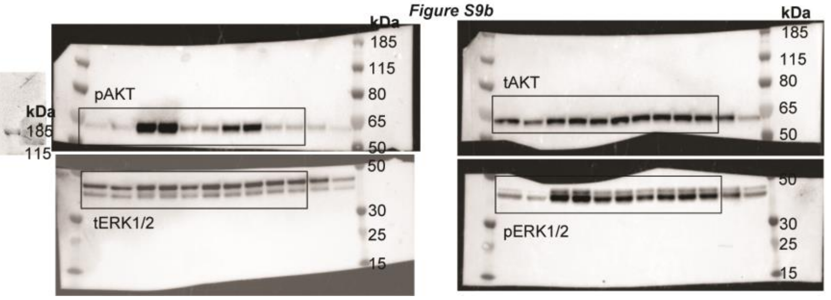

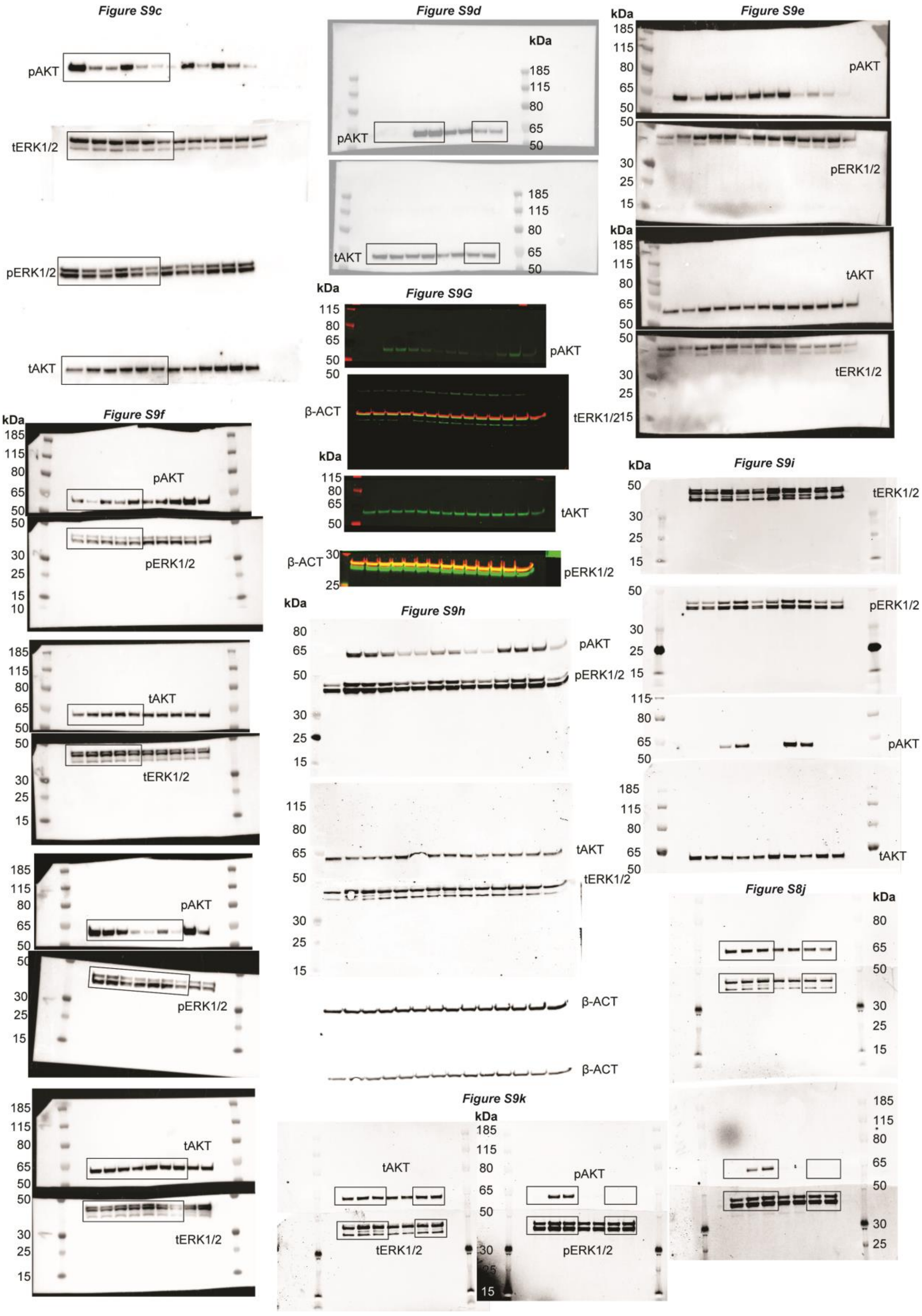
Figure S9c
pAKT
tERK1/2
pERK1/2
tAKT
Figure S9d
kDa
185
115
80
65
50
pAKT
tAKT
Figure S9e
pERK1/2
tERK1/2
Figure S9G
β-ACT
Figure S9f
Figure S9h
Figure S9i
Figure S8j
Figure S9k

Supplementary Figure 10. Unedited western blots. Unedited western blots of *Figure 1b, 1e, 2c, 2e* (=overlay of protein blots with markers); *Figure 2g, 4c, 4d, 8b, 8e, 5c, 8g, 6b, 6d, 7a, 7b, S1c, S1d, S1f, S3c, S5g, S6a, S6c, S7a, S7c, S7d, S9a-S9k.* Blots are displayed with marker and with the selected area with specific proteins.
