# Supplementary material for: Cardiac progenitor cell-derived extracellular vesicles promote angiogenesis through both associated- and co-isolated proteins
Source: Commun Biol. 2023 Aug 1;6:800. doi: 10.1038/s42003-023-05165-7 (PMC10393955; doi:10.1038/s42003-023-05165-7)
Supplement: Supplementary file 2 — Description of Additional Supplementary Files [file 42003_2023_5165_MOESM2_ESM.pdf]

Description of Additional Supplementary Files

File name: Supplementary Data 1

Description: Proteomics data are deposed in PRIDE Graph data are present in Supplementary Data file
