# Supplementary material for: Cardiac progenitor cell-derived extracellular vesicles promote angiogenesis through both associated- and co-isolated proteins
Source: Commun Biol. 2023 Aug 1;6:800. doi: 10.1038/s42003-023-05165-7 (PMC10393955; doi:10.1038/s42003-023-05165-7)
Supplement: Supplementary file 4 — Reporting Summary [file 42003_2023_5165_MOESM4_ESM.pdf]

nature portfolio

Corresponding author(s): Marieke T. Roefs

Last updated by author(s): Jul 6, 2023

Reporting Summary

Nature Portfolio wishes to improve the reproducibility of the work that we publish. This form provides structure for consistency and transparency in reporting. For further information on Nature Portfolio policies, see our Editorial Policies and the Editorial Policy Checklist.

Statistics

For all statistical analyses, confirm that the following items are present in the figure legend, table legend, main text, or Methods section.

n/a	Confirmed	
☐	☒	The exact sample size (n) for each experimental group/condition, given as a discrete number and unit of measurement
☐	☒	A statement on whether measurements were taken from distinct samples or whether the same sample was measured repeatedly
☐	☒	The statistical test(s) used AND whether they are one- or two-sided<br>*Only common tests should be described solely by name; describe more complex techniques in the Methods section.*
☒	☐	A description of all covariates tested
☐	☒	A description of any assumptions or corrections, such as tests of normality and adjustment for multiple comparisons
☐	☒	A full description of the statistical parameters including central tendency (e.g. means) or other basic estimates (e.g. regression coefficient) AND variation (e.g. standard deviation) or associated estimates of uncertainty (e.g. confidence intervals)
☐	☒	For null hypothesis testing, the test statistic (e.g. F, t, r) with confidence intervals, effect sizes, degrees of freedom and P value noted<br>*Give P values as exact values whenever suitable.*
☒	☐	For Bayesian analysis, information on the choice of priors and Markov chain Monte Carlo settings
☐	☒	For hierarchical and complex designs, identification of the appropriate level for tests and full reporting of outcomes
☒	☐	Estimates of effect sizes (e.g. Cohen's d, Pearson's r), indicating how they were calculated

Our web collection on statistics for biologists contains articles on many of the points above.

Software and code

Policy information about availability of computer code

Data collection	All (phospho)proteomics raw data were searched in MaxQuant (v_1.6.10.43) against a the SwissProt human reference proteome database (containing 20,381 proteins and downloaded from Uniprot on March 2021). Spectra were searched using MaxQuant's built-in Andromeda search engine. Data was analyzed using Perseus software (v_1.6.14)
Data analysis	Gene ontology analysis were done using PANTHER with the human proteome as background gene set. All (phospho)proteomic plots were generated using common R packages.

For manuscripts utilizing custom algorithms or software that are central to the research but not yet described in published literature, software must be made available to editors and reviewers. We strongly encourage code deposition in a community repository (e.g. GitHub). See the Nature Portfolio guidelines for submitting code & software for further information.

Data

Policy information about availability of data

All manuscripts must include a data availability statement. This statement should provide the following information, where applicable:

- Accession codes, unique identifiers, or web links for publicly available datasets
- A description of any restrictions on data availability
- For clinical datasets or third party data, please ensure that the statement adheres to our policy

The (phospho)proteomic datasets generated and analysed during the current study are available in the ProteomeXchange Consortium via the PRIDE repository, and can be accessed through the identifier PXD030779.

Research involving human participants, their data, or biological material

Policy information about studies with human participants or human data. See also policy information about sex, gender (identity/presentation), and sexual orientation and race, ethnicity and racism.

Reporting on sex and gender	n/a
Reporting on race, ethnicity, or other socially relevant groupings	n/a
Population characteristics	n/a
Recruitment	n/a
Ethics oversight	n/a

Note that full information on the approval of the study protocol must also be provided in the manuscript.

Field-specific reporting

Please select the one below that is the best fit for your research. If you are not sure, read the appropriate sections before making your selection.

☒ Life sciences ☐ Behavioural & social sciences ☐ Ecological, evolutionary & environmental sciences

For a reference copy of the document with all sections, see nature.com/documents/nr-reporting-summary-flat.pdf

Life sciences study design

All studies must disclose on these points even when the disclosure is negative.

Sample size	Sample size determination was performed based on effects observed in previous experiments. We used biological triplicates to show true effect in our in vitro assays.
Data exclusions	No data was excluded for analysis in this study.
Replication	All attempts for data replication (of e.g. western blot analyses of cell activation) were successful in this study, and are included in the supplemental figures.
Randomization	Randomization was applied when possible, such as applying a random treatment schedule within tissue culture plates and using random and variable order of extracellular vesicle isolation using the same size-exclusion chromatography column.
Blinding	Authors were blinded when possible, such as when analyzing in vitro scratch assay results.

Reporting for specific materials, systems and methods

We require information from authors about some types of materials, experimental systems and methods used in many studies. Here, indicate whether each material, system or method listed is relevant to your study. If you are not sure if a list item applies to your research, read the appropriate section before selecting a response.

Materials & experimental systems

n/a	Involved in the study
☐	☒ Antibodies
☐	☒ Eukaryotic cell lines
☒	☐ Palaeontology and archaeology
☒	☐ Animals and other organisms
☒	☐ Clinical data
☒	☐ Dual use research of concern
☒	☐ Plants

Methods

n/a	Involved in the study
☒	☐ ChIP-seq
☒	☐ Flow cytometry
☒	☐ MRI-based neuroimaging

Antibodies

Antibodies used	rabbit anti-phospho-ERK (Phospho-p44/p42 MAPK (Erk1/2) (Thr202/Tyr204, Cell signaling Technology, 1:1,000), rabbit anti-ERK (p44/p42 (Erk1/2), Cell Signaling Technology, 1:1,000), rabbit anti-phospho-AKT (Ser473, Clone D9E, Cell Signaling Technology, 1:1,000), rabbit anti-AKT (Cell Signaling Technology, 1:1,000), mouse anti-CD63 (Abcam, 1:1,000), rabbit anti-CD9 (Abcam, 1:1,000), mouse anti-ALIX (Thermo Scientific, MA1-83977), rabbit anti-Calnexin (GeneTex, GTX 101676, 1:1,000), mouse anti-β-actin (Sigma, 1:5,000), rabbit anti-TSG101 (Abcam, 1:1,000), mouse anti-Syntenin-1 (Origene, TA504796, 1:1,000), rabbit anti-Annexin A1 (Abcam, ab214486, 1:1,000)), goat anti-Nidogen-1 (R&D Systems, AF2570), goat anti-PAPP-A (R&D Systems, AF2487), mouse anti-PAPP-A (Hytest, 4PD4) and mouse anti-CD81 (clone B-11, Santa Cruz, 1:1,000). Secondary antibodies included Alexa680-conjugated goat anti-mouse (Thermo Fisher Scientific, 1:7,500) and IRG800-conjugated goat anti-rabbit (LI-COR Biosciences, 1:7,500).
Validation	Antibodies were validated by vendors.<br>https://www.cellsignal.com/products/primary-antibodies/phospho-p44-42-mapk-erk1-2-thr202-tyr204-antibody/9101<br>https://www.cellsignal.com/products/primary-antibodies/p44-42-mapk-erk1-2-antibody/9102<br>https://www.cellsignal.com/products/primary-antibodies/phospho-akt-ser473-antibody/9271<br>https://www.cellsignal.com/products/primary-antibodies/akt-antibody/9272<br>https://www.thermofisher.com/antibody/product/Alix-Antibody-clone-3A9-Monoclonal/MA1-83977<br>https://www.genetex.com/Product/Detail/Calnexin-antibody-N3C2-Internal/GTX101676<br>https://www.abcam.com/TSG101-antibody-ab30871.html<br>https://www.origene.com/catalog/antibodies/primary-antibodies/ta504796/syntenin-sdcbp-mouse-monoclonal-antibody-clone-id-oti2h6<br>https://www.abcam.com/annexin-a1anxa1-antibody-epr19342-ab214486.html<br>https://www.scbt.com/sv/p/cd81-antibody-b-11<br>https://shop.hytest.fi/product/papp-human-antibody<br>https://www.rndsystems.com/products/human-pappalysin-1-papp-a-antibody_af2487<br>https://www.rndsystems.com/products/human-nidogen-1-entactin-antibody_af2570

Eukaryotic cell lines

Policy information about cell lines and Sex and Gender in Research

Cell line source(s)	Cardiac progenitor cells (CPCs, donor HFH070809) were obtained from human fetal hearts at Leiden University Medical Center, The Netherlands.<br>Human microvascular endothelial cells (HMEC-1, male) were obtained from ATCC.<br>Human epithelial SKOV-3 ovarian adenocarcinoma cells (female) were obtained from ATCC.
Authentication	Sca-1+ CPC lines used in this study were authencicated before by Smits et al.
Mycoplasma contamination	All cell lines were negatively tested for mycoplasma
Commonly misidentified lines (See ICLAC register)	n/a
